# Interfacial Electronic Modulation of Dual-Monodispersed Pt–Ni_3_S_2_ as Efficacious Bi-Functional Electrocatalysts for Concurrent H_2_ Evolution and Methanol Selective Oxidation

**DOI:** 10.1007/s40820-023-01282-4

**Published:** 2024-01-11

**Authors:** Qianqian Zhao, Bin Zhao, Xin Long, Renfei Feng, Mohsen Shakouri, Alisa Paterson, Qunfeng Xiao, Yu Zhang, Xian-Zhu Fu, Jing-Li Luo

**Affiliations:** 1 Shenzhen Key Laboratory of Energy Electrocatalytic Materials, Shenzhen Key Laboratory of Polymer Science and Technology, Guangdong Research Center for Interfacial Engineering of Functional Materials, College of Materials Science and Engineering, Shenzhen University, Shenzhen, 518060 People’s Republic of China; 2 Canadian Light Source Inc., Saskatoon, SK S7N 0X4 Canada; 3 Instrumental Analysis Center of Shenzhen University (Lihu Campus), Shenzhen University, Shenzhen, 518055 People’s Republic of China

**Keywords:** Dual-monodispersed heterostructure, Electronic interactive modulation, Reaction mechanism, Methanol oxidation reaction, Hydrogen generation

## Abstract

Constructing the efficacious and applicable bi-functional electrocatalysts and establishing out the mechanisms of organic electro-oxidation by replacing anodic oxygen evolution reaction (OER) are critical to the development of electrochemically-driven technologies for efficient hydrogen production and avoid CO_2_ emission. Herein, the hetero-nanocrystals between monodispersed Pt (~ 2 nm) and Ni_3_S_2_ (~ 9.6 nm) are constructed as active electrocatalysts through interfacial electronic modulation, which exhibit superior bi-functional activities for methanol selective oxidation and H_2_ generation. The experimental and theoretical studies reveal that the asymmetrical charge distribution at Pt–Ni_3_S_2_ could be modulated by the electronic interaction at the interface of dual-monodispersed heterojunctions, which thus promote the adsorption/desorption of the chemical intermediates at the interface. As a result, the selective conversion from CH_3_OH to formate is accomplished at very low potentials (1.45 V) to attain 100 mA cm^−2^ with high electronic utilization rate (~ 98%) and without CO_2_ emission. Meanwhile, the Pt–Ni_3_S_2_ can simultaneously exhibit a broad potential window with outstanding stability and large current densities for hydrogen evolution reaction (HER) at the cathode. Further, the excellent bi-functional performance is also indicated in the coupled methanol oxidation reaction (MOR)//HER reactor by only requiring a cell voltage of 1.60 V to achieve a current density of 50 mA cm^−2^ with good reusability.

**Supplementary Information:**

The online version contains supplementary material available at 10.1007/s40820-023-01282-4.

## Highlights

- The well-conceived Pt–Ni_3_S_2_ heteronanocrystals with dual-monodispersed characteristics are synthesized through interfacial electronic modulation.
- The asymmetrical charge distribution at Pt–Ni_3_S_2_ hetero-interface results in the formation of high-valent Ni sites and negatively-charged Pt^*δ*−^.
- It eventually accelerates water dissociation and achieves the steady concurrent generation of value-added formate and hydrogen.

**Supplementary Information:**

The online version contains supplementary material available at 10.1007/s40820-023-01282-4.

## Graphical abstract

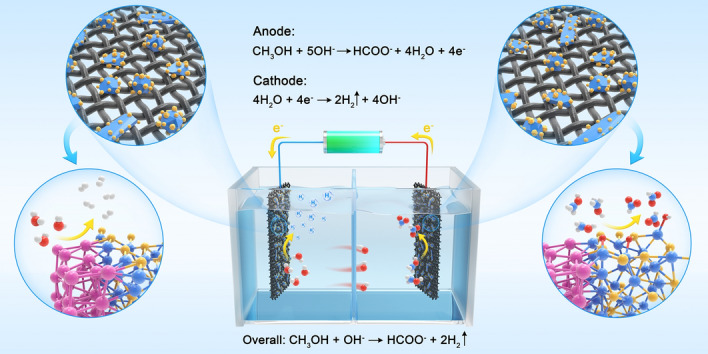

## Introduction

The high dependence on fossil fuels worldwide has resulted in serious environmental pollution and climate change, leading to energy crises. It is imperative for us to conform to the development of the times, phase out fossil fuels, and strive to reduce the usage of non-renewable energy by developing clean energy [1, 2]. Hydrogen has apparent advantages over other energy sources because of its extensive origins, high combustion calorific value, and eco-friendly production possibilities. However, conventional large-scale hydrogen production technologies mainly include coal gasification and steam reforming, which result in massive emissions of environment-unfriendly CO_2_ gas [3]. Compared to other methods, water-splitting appears to be a perspective and environmentally friendly method for hydrogen production. Despite this advantage, the hydrogen production efficiency is further restricted by oxygen evolution reaction (OER)’s kinetics at the anodes during water splitting, which causes the high overall energy consumption during electrolysis. At the moment, noble metal Pt-based catalysts are outstanding catalysts for water-splitting because of their perfect adsorption properties in volcanic type activity trends and several representative electrocatalytic qualities including excellent electrochemical activity, chemical stability and corrosion resistance. However, the price of precious metals is expensive due to their extreme rarity and high demand. As a result, it is believed that monometallic catalysts based on noble metals are less likely to be used in industrial production [4–6].

Hence, developing bi-functional catalysts with low platinum load recently becomes an effective strategy for hydrogen evolution via organic-water co-electrolysis, which can not only decrease energy consumption of hydrogen evolution by reducing the cell voltage, but also concurrently obtain value-added organic chemical products at the anode, making this reaction academically and industrially significant [7]. Formic acid has a higher market value compared to methanol, it is an important intermediate in the chemical industry with higher value (> 539 €/ton) and commonly used for synthesizing various fine chemicals. Methanol has received wide attention in comparison to other small organic compounds including urea, ethanol, glycerol, amine, formaldehyde [8, 9], hydrazine hydrate [10, 11], furfural [12, 13], and 5-hydroxymethylfurfural owing to its strong oxidation reactivity, high water solubility, and affordable pricing (about 350 €/ton) [14–16]. Electro-oxidation of methanol to formic acid (or formate) should be a feasible method, because it can not only achieve the selective upgrading reactions for value-added chemicals but also reduce the working voltage of water electrolysis to achieve the energy-saving hydrogen production [17, 18]. Single-component metal sulfide has shown specific potential in methanol selective oxidation and it has reached a high level at present. However, their performance remains to be improved compared with the commercial electrocatalysts in methanol–water co-electrolysis as a bi-functional catalyst [19–22]. Although the commercially used platinum catalysts have good performance in hydrogen production, they have the drawbacks of low earth content. Further, as a bi-functional catalyst, the efficiency of such co-electrolysis for formate-H_2_ co-production is limited by CO poisoning during the methanol oxidation reaction (MOR), preventing high current densities from steady working overtime [14, 23]. In addition, the possible production of CO_2_ gas is also very undesirable under the current global policy of low carbon economy. Therefore, there are still many challenges in developing bi-functional catalysts [24].

Reducing the loading of precious metal Pt and combining it with chalcogenide multiphase compounds may be an effective strategy to further decrease the voltage of water electrolysis and boost the efficiency of hydrogen production. By now, some previous works have found that various heterojunction nanomaterials formed by the combination of precious metal Pt and chalcogenide compounds have a significant effect on enhancing the activity of catalysts for HER, OER, CO_2_RR, and other catalytic reactions [25, 26]. The key reasons are the synergistic effects of the constructed interface which was generated from the contacting region due to influence each other to increase the catalyst activity significantly [27–29]. Nowadays, the design of low platinum loading catalysts typically includes some strategies. Among them, platinum nanoparticles are integrated into specific carrier to enhance the dispersibility and mass activity of platinum [30–32]. Moreover, some atomically distributed Pt is further stabilized by adhering to the carrier's surface with carefully designed vacancies or coordination groups to maximize the platinum's activity and usage efficiency [33–35]. Furthermore, it is possible to increase the activity of the platinum component by combining the precious metal with other transition metals [36–40]. However, the active sites of noble metal platinum could not be highly exposed owing to low specific surface area and poor dispersibility of platinum-chalcogenide nanoheterojunction materials, which still hinder the critical progress on electrocatalytic methanol–water co-electrolysis [25–27, 37, 41]. In additional, Pt nanoparticles are susceptible to poisoning by adsorbed intermediates (i.e., CO), resulting in activity deterioration and unsatisfactory hydrogen production efficiency when acting as bi-functional catalysts for methanol–water co-electrolysis [14, 42, 43]. Therefore, there are still some challenges in developing bi-functional catalysts with low Pt loading, good dispersion of nanoscale Pt particles to highly promote the methanol oxidation activity and hydrogen production efficiency.

**Scheme 1:**
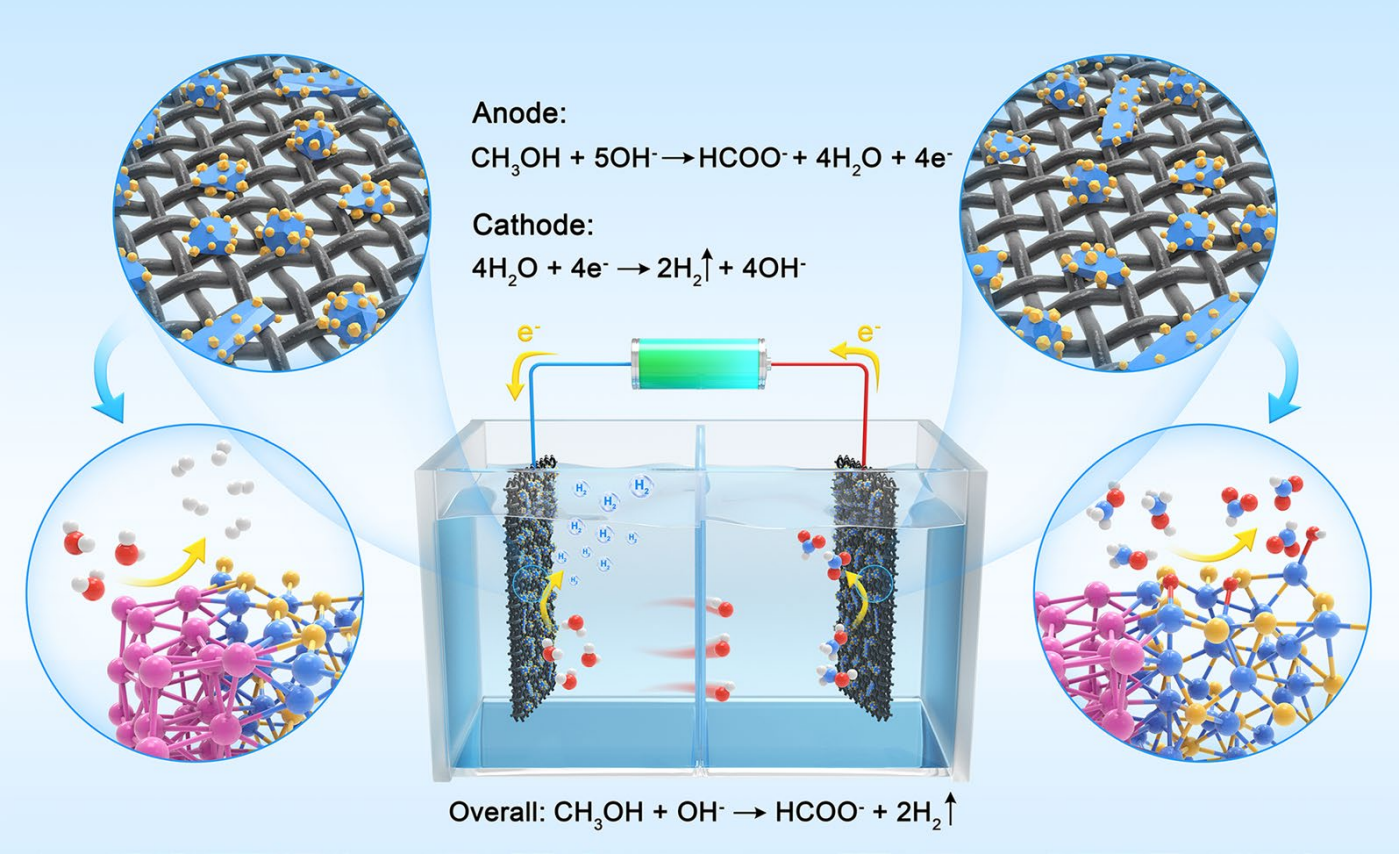
Diagram of the as-synthesized *DMD* Pt–Ni_3_S_2_ *HNCs* used as electrodes for MOR and HER

Herein, the dual-monodispersed Pt–Ni_3_S_2_ hetero-nanocrystals (“*DMD* Pt–Ni_3_S_2_ *HNCs*”) were synthesized as catalysts by modifying the electronic structure of the catalysts through interfacial engineering. The secondary monodispersed Pt nanocrystals with a size of ~ 2 nm were heterogeneously bonded with the primary monodispersed Ni_3_S_2_ nanocrystals with a size of ~ 9.6 nm by chemical interaction through hot injection, thus leading to the formation of “*DMD* Pt–Ni_3_S_2_ *HNCs*” with highly abundant lattice defects as charge transport channels at the dense heterogeneous interface. These heterojunction interfaces can further induce abundant active sites to enhance the catalytic activity, which is valuable for studying the mechanism of methanol oxidation and hydrogen reaction. The as-synthesized “*DMD* Pt–Ni_3_S_2_ *HNCs*” can selectively catalyze CH_3_OH to more valuable chemical (formate) at 1.45 V (vs. RHE) to achieve 100 mA cm^−2^ with high Faradaic efficiency (FE > 98%). Notably, attributed to the fine interfacial electronic modulation and the dual-monodispersed features, the “*DMD* Pt–Ni_3_S_2_ *HNCs*” exhibited superior bi-functional performance, only requiring a low overpotential of 61 mV to run the HER at 10 mA cm^−2^ in 1.0 mol L^−1^ KOH. Experimental investigation and theoretical studies clarify that the existence of Pt nanocrystals on the surface of “*DMD* Pt–Ni_3_S_2_ *HNCs*” is playing an imperative role, in promoting and stabilizing high-valent Ni sites and further accelerates H* conversion and H_2_ desorption in hydrogen evolution. Through strong electronic interaction provided by the interface of high dispersion of Pt nanocrystals and Ni_3_S_2_ nanocrystals, the “*DMD* Pt–Ni_3_S_2_ *HNCs*” with bilateral synergetic active sites show excellent performance and selectivity for the application of bi-functional electrocatalysts, which eventually achieve the concurrent generation of value-added formate and hydrogen, as illustrated in Scheme 1. This work provides an approach towards rational design of efficient bi-functional electrocatalysts through precise construction of interfaces.

## Experimental Methods

### Synthesis of Pt–Ni_3_S_2_ Catalyst

The one-pot solution-based syntheses were performed using a standard *Schlenk* vacuum line technique under argon atmosphere. In a standard synthesis, 2 mmol (0.5138 g) Ni(acac)_2_ and OLA (20 mL) were fully dissolved in a round-bottom *Schlenk* flask (100 mL) at room temperature. The flask was degassed under vacuum at 80 °C for 0.5 h to remove oxygen and other low-boiling-point organic solvent. Subsequently, the reaction was programmed to be 220 °C with a ramp of 5 °C min^−1^ after backfilling with Ar in oil bath. At the same time, 1 mmol (0.2184 g) DPDS and 3 mL OLA, 0.1 mmol (0.04 g) Pt(acac)_2_ and 1 mL OLA was separately mixed in a glass vial, then preheated to 80 °C on a hot plate to form a clear solution. When the flask reaches 220 °C, the diphenyl disulfide solution was injected into the metal solution by syringe. After injection, the temperature drops to 210–215 °C and the reaction was allowed to maintain at 215 °C for 10 min with continuous stirring. After 10 min dwelling for the growth of Ni_3_S_2_ NPs, the acetylacetone platinum solution was further injected into the Ni_3_S_2_ dispersion by syringe. With constant stirring, the reaction was allowed to continue at that temperature for 5 min. After stopping the reaction, the flask was taken out of the oil bath and allowed to naturally cool to room temperature. The product was dissolved in toluene and the solution was centrifuged at 12,000 rpm during 10 min for nanoparticles separation. Finally, the as-synthesized Pt–Ni_3_S_2_ nanocrystals were thoroughly purified by multiple precipitation and re-dispersion steps using toluene and isopropanol.

### Electrocatalytic Experiments

Electrochemical measurements were achieved by CHI760E (CH Instruments, Inc. Shanghai, China) electrochemical analyzer at room temperature with standard three-electrode system. The sample was chosen as the working electrode and a saturated Ag/AgCl (sat. KCl) and the platinum foil were chosen as the reference electrode and the counter electrode, respectively. Two-electrode system using Pt–Ni_3_S_2_ sample as both the cathode and anode was utilized to estimate the performance of water splitting. All the potentials were converted to the reversible hydrogen electrode (RHE) according to the Nernst equation, the conversion equation is *E*(RHE) = *E*(Ag/AgCl) + 1.0205 V (Fig. S5). 20 cycles of CVs cans were conducted for every working electrode with electrocatalyst before the data collection. The linear sweep voltammetry (LSV) curves were recorded at a scan rate of 5 mV s^−1^ without *iR*-correction. Electrochemical impedance spectroscopy (EIS) was undertaken at 1.49 V (vs. RHE) with AC amplitude of 5 mV and frequency range of 0.01–100 kHz. Moreover, the cyclic voltammetry (CV) curves were collected in the potential range from 1.02 to 1.12 V (vs. RHE) with the scan rates of 20, 40, 60, 80, 100 and 120 mV s^−1^ to obtain the double-layer capacitance of electrocatalyst. Ion Chromatography (CIC-D120, SHENGHAN, China) was employed to identify and quantify the value-added chemical product (formate). The generated H_2_ in the cathode compartment were determined by gas chromatography (5977B MSD, Agilent Technologies). At least three chromatographic trace curves were collected for statistical analysis.

### Catalyst Characterization

The morphological information of Pt–Ni_3_S_2_ catalyst was characterized by field emission transmission electron microscopy (FETEM JEM-F200) including SAED and EDS elemental mapping functions. Crystallographic and purity information on Pt–Ni_3_S_2_ catalyst were obtained by X-ray powder diffraction (XRD, RIGAKU Smartlab), The elemental components of Pt–Ni_3_S_2_ was studied by X-ray photoelectron spectroscopy (XPS, ESCALAB 250Xi), X-ray absorption fine structure (XAFS) spectra XANES, FT-EXAFS and WT-EXAFS, and Inductively coupled plasma-mass spectrometry (ICP-MS, PerkinElmer NexION 300X).

### Density Functional Theory Calculation

Density functional theory (DFT) calculations were performed to uncover the mechanism of selective oxidation of methanol and hydrogen evolution. The first principle calculations are performed to reveal the mechanism by using the Vienna ab initio simulation package [44, 45]. The program has the projected enhancement wave pseudopotential [46] and the generalized gradient approximation of Perdew, Burke and Enzzerhof (PBE) exchange correlation functional [47], which is used to optimize the structure and obtain the free energy of all structures.

Based on the experimental outcomes, the lattice parameters of Ni_3_S_2_ and Pt were used for DFT calculations, i.e., *a* = 11.54660 Å, *b* = 8.11980 Å, *c* = 22.10230 Å, *α* = *β* = 90°, *γ* = 90.8935° for Ni_3_S_2_ and *a* = *b* = 11.2480 Å, *c* = 19.59200 Å, *α* = *β* = 90.00°, *γ* = 120.00° for Pt. According to the experimental observation, the heterogeneous interfaces are mostly associated with Ni_3_S_2_ (110) and Pt (111) facets, thus suggesting them as a key reaction point for DFT studies. So, the lattice parameters of Pt–Ni_3_S_2_ were used for DFT calculations, i.e., *a* = 8.27790 Å, *b* = 20.07430 Å, *c* = 25.68030 Å, *α* = *β* = *γ* = 90°. In addition, the cutoff energy of the plane waves basis set is 500 eV in the adsorption energy calculation, the electron self-consistent iteration was set to 10^−5^ eV and the positions of all atoms were completely relaxed until the residual force per atom was below 0.05 eV Å^−1^. A vacuum layer of 15 Å was applied along the *z*-direction to avoid periodic interactions.

## Results and Discussion

### Synthesis and Characterization

**Fig. 1:**
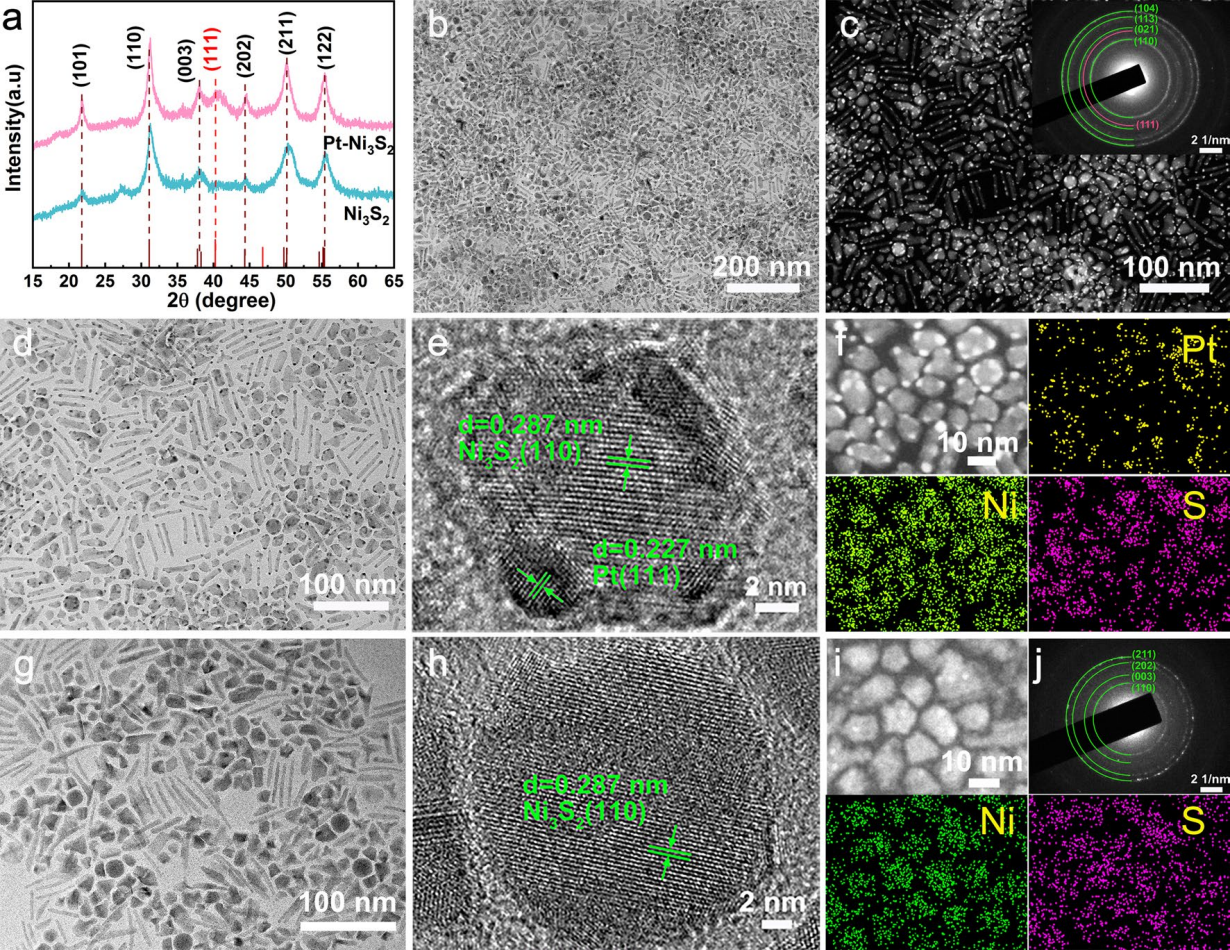
Composition characterizations and morphology of the Pt–Ni_3_S_2_ and Ni_3_S_2_ nanocrystals. **a** XRD results; **b** TEM images, **c** HAADF-STEM image and SAED pattern (inset), **d**–**e** HRTEM images and **f** EDS elemental mappings of Pt–Ni_3_S_2_; **g**–**h** HRTEM, **i** EDS mapping, and **j** SAED pattern of Ni_3_S_2_ nanocrystals

The “*DMD* Pt–Ni_3_S_2_ *HNCs*” (or Pt–Ni_3_S_2_ for short in the following paragraphs) catalyst was fabricated through one-step hot injection method, as illustrated in Scheme S1. In the study of the crystal growth process of nanomaterials, the most fundamental classical thermodynamic theory is the Gibbs–Curie–Wulff crystal growth theory [48]. When the Pt precursor is introduced into the reaction system with a large number of already formed Ni_3_S_2_ nanoparticles, the free single Pt atoms originated from precursor are not stable so that they will tend to attach onto the surface of already existed Ni_3_S_2_ nanoparticles in order to reduce their surface free energies [48]. As the time increases, the Pt nuclei will form and the nanocrystals growth will occur, the process of which follows the Ostwald ripening [49, 50] Eventually, it reaches a minimum size of nuclear stabilization and grows into small platinum particle heterogenized onto Ni_3_S_2_ nanoparticles. XRD patterns (Fig. 1a) show the phase results of the products without introducing platinum precursor into the synthesis, the diffraction peaks at 31.10°, 50.12°, and 55.16° match well with the (110), (211), and (122) planes of Ni_3_S_2_ (PDF # 00-044-1418). Furthermore, it was evident that the peaks at 40.25° belong to the (111) planes of Pt (PDF # 01-087-0647) after adding platinum precursor in the hot injection procedure. It represents that Pt-rich second-phase material has been successfully introduced into the catalyst. The weight percentages of Pt–Ni_3_S_2_ were obtained from ICP-MS. The amount of Pt loading in Pt–Ni_3_S_2_ was controlled to 9 wt% as shown in Table S1, which well matches the ratio of added elements. The microscopic structures of Pt–Ni_3_S_2_ were investigated by TEM. As illustrated in Figs. 1b–d and S1, S2, the monodispersed Pt nanocrystals (~ 2 nm) are uniformly fixed on the primary Ni_3_S_2_ nanocrystals. Notably, TEM image (Fig. 1d) obviously displays monodispersed Pt–Ni_3_S_2_ nanocrystals with an average size of ~ 9.6 nm, which demonstrated that Ni_3_S_2_ nanocrystals possess the shape anisotropy structure [51]. We have also discovered that the size of Pt–Ni_3_S_2_ was inhibited due to the growth of Pt nanocrystals. Rarely, both Pt nanocrystals and Ni_3_S_2_ nanocrystals exhibit well uniform dispersion properties, but also the heterojunction formed by the combination of the Pt nanocrystals and Ni_3_S_2_ nanocrystals can also maintain the dual-monodispersed nanoheterostructures, which provides the possibility to increase the catalyst surface area and expose more active sites. Moreover, dark-field TEM images (Fig. 1c) further confirm the excellent dispersion of Pt–Ni_3_S_2_, which are expected to considerably increase the specific surface area. The Pt–Ni_3_S_2_ was indicated by SAED (an inset in Fig. 1c) patterns, in which the lattice planes’ spacings indicated by the diffraction rings correspond well with PDF # 00-044-1418 (Ni_3_S_2_) and 01-087-0647 (Pt). All these results indicate that the Pt–Ni_3_S_2_ have the ultra-small nanocrystals size and the Pt-rich vertices, which can considerably expose the surface active sites of the catalyst to favor rapid mass and electron diffusion. The magnified TEM image of Fig. 1d–e further revealed the formation of Pt–Ni_3_S_2_. The lattice planes of 0.227 nm and 0.287 nm, respectively, correspond to the (111) facet of metallic Pt and the (110) facet of Ni_3_S_2_, which indicates that the Pt and Ni_3_S_2_ co-exist in the particle rather than separated grains. Among Pt and Ni_3_S_2_, there are a large number of hetero-interfaces, which can generate a large number of lattice defects to stimulate the formation of abundant active sites [52, 53]. The EDS mapping images in Fig. 1f explicitly manifest evenly distributions of all three elements within the Pt–Ni_3_S_2_. Figures 1g and S1 present the clear structures of single Ni_3_S_2_ nanocrystal plates with the size of ~ 15 nm. Furthermore, the extra HRTEM image demonstrates a substantial amount of clear lattice fringes corresponded to the (110) facets of Ni_3_S_2_ in Fig. 1h. The matching EDS elemental mappings confirm (Fig. 1i) the existence of Ni and S. Notably, various lattice planes of highly dispersed Ni_3_S_2_ nanocrystals (PDF # 00-044-1418) agree well with the distinct diffraction rings shown in Fig. 1j.

**Fig. 2:**
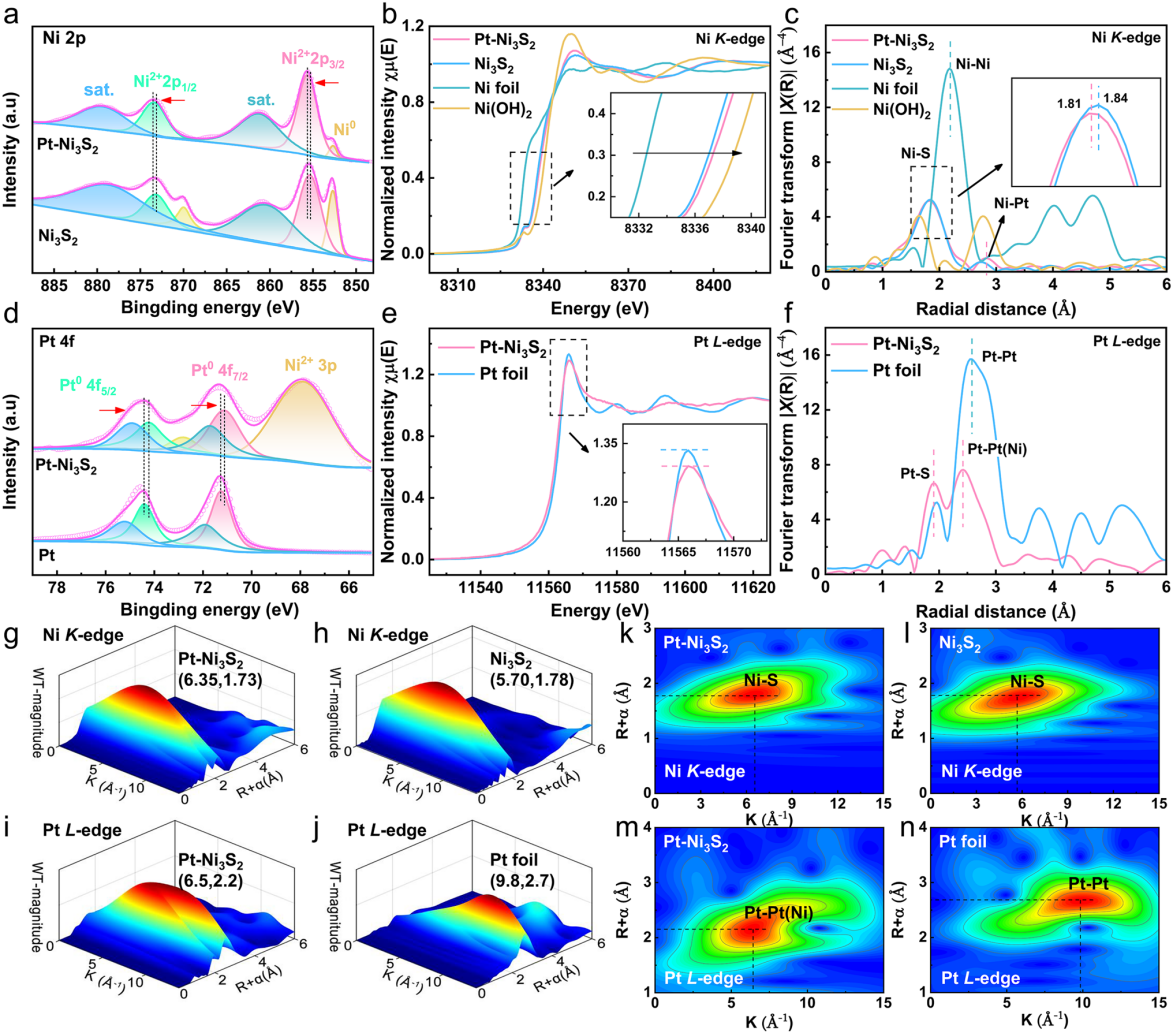
High-resolution XPS spectra and XAFS spectra of Pt–Ni_3_S_2_ and Ni_3_S_2_. **a** Ni 2*p* XPS spectra of Pt–Ni_3_S_2_ and Ni_3_S_2_; **b** XANES of Ni *K*-edge spectra for Pt–Ni_3_S_2_, Ni_3_S_2_, Ni foil, and Ni(OH)_2_; **c** FT-EXAFS of Ni *K*-edge spectra in R space for Pt–Ni_3_S_2_, Ni_3_S_2_, Ni foil, and Ni(OH)_2_; **d** Pt 4*f* XPS spectra of Pt–Ni_3_S_2_ and Ni_3_S_2_; **e** XANES of Pt *L*-edge spectra for Pt–Ni_3_S_2_, Pt foil; **f** FT-EXAFS of Pt *L*-edge spectra in R space for Pt–Ni_3_S_2_, Pt foil; WT-EXAFS of Ni *K*-edge spectra for **g** Pt–Ni_3_S_2_, **h** Ni_3_S_2_ and the corresponding 2D spectra for **k** Pt–Ni_3_S_2_, **l** Ni_3_S_2_; WT-EXAFS of Pt *L*-edge spectra for **i** Pt–Ni_3_S_2_, **j** Pt foil and the corresponding 2D spectra for **m** Pt–Ni_3_S_2_, **n** Pt foil

For more details on surface chemical states between Pt and Ni_3_S_2_ catalysts was obtained by resorting to the XPS. In Fig. 2a, Ni 2*p* XPS spectra of Pt–Ni_3_S_2_ presents two visible peaks at 873.4 and 855.64 eV that should be allocated to Ni 2*p*_1/2_ and Ni 2*p*_3/2_ orbits of Ni^2+^ [54]. After Pt nanocrystals is anchored to Ni_3_S_2_ nanocrystals, there was a positive shift in the binding energy of Ni 2*p*_1/2_ and Ni 2*p*_3/2_ of Ni^2+^ in Pt–Ni_3_S_2_, indicating that the platinum atom influences the electronic structure of the element Ni in the Pt–Ni_3_S_2_ to result in a higher Ni valence state. In addition, the lower content of Ni^0^ species of Pt–Ni_3_S_2_ compared with Ni_3_S_2_ may be associated with lightly absorption of energy from Pt nanocrystals placed on the Pt–Ni_3_S_2_ [54]. Furthermore, XPS spectrum in O 1*s* orbital (Fig. S3b) shows that the surface of Pt–Ni_3_S_2_ and Ni_3_S_2_ contains some OH^−^ and water species. It is easy to adsorb a small amount of oxygen-containing species due to the small particle size of the catalyst, which is also very conducive to the activation of methanol [55, 56]. The electronic state and atomic environment of Ni atoms in Pt–Ni_3_S_2_, Ni_3_S_2_, Ni foil, and Ni(OH)_2_ are as well as further characterized by X-ray absorption near edge structure (XANES) in Fig. 2b. The white line intensity of Ni *K*-edge in Pt–Ni_3_S_2_ nanocrystals are located between Ni foil and Ni(OH)_2_, which indicates that the valance state of the element Ni in the Pt–Ni_3_S_2_ nanocrystals is positive but less than the valance state of Ni(OH)_2_ [53]. Furthermore, the absorption edge of Ni in Pt–Ni_3_S_2_ (Fig. 2b) is slightly higher than that in Ni_3_S_2_, which is similar with the XPS analysis' result that Ni in Pt–Ni_3_S_2_ has a higher valence than Ni_3_S_2_. Figure 2c shows central atoms' radial distribution function by FT-EXAFS results in R space for Pt–Ni_3_S_2_, Ni_3_S_2_, Ni foil, and Ni(OH)_2_. The prominent peaks at around 1.82 and 2.80 Å for Pt–Ni_3_S_2_ correspond to Ni–S bond and Ni–Pt bond [53, 57]. The Ni–S bond length (1.81 Å) in Pt–Ni_3_S_2_ is a little shorter than the one in Ni_3_S_2_ (1.84 Å) due to the higher oxidation state of the element Ni in Pt–Ni_3_S_2_, indicating a more stable Ni–S connection [25]. In addition, the XPS of S 2*p* spectrum (Fig. S3a) presents two prominent peaks in Pt–Ni_3_S_2_ at 163.06 and 161.62 eV, designated as S 2*p*_1/2_ and S 2*p*_3/2_ orbitals of S 2*p*, respectively. Compared to Ni_3_S_2_, we found that two prominent peaks have a positive shift, indicating a higher S valence state in Pt–Ni_3_S_2_.

Meanwhile, the electronic states around the Pt atom in Pt–Ni_3_S_2_ were verified by XPS, XANES and EXAFS measurements. First, Pt 4*f* XPS spectra of Pt–Ni_3_S_2_ (Fig. 2d) shows that two XPS peaks for Pt^0^ 4*f*_7/2_ and Pt^0^ 4*f*_5/2_ of Pt–Ni_3_S_2_ at 71.119 and 74.219 eV indicate a negative shift in comparison with pristine Pt foil, suggesting the presence of negatively-charged Pt atoms (Pt^*δ*−^) of Pt in Pt–Ni_3_S_2_ than that in Pt foil [32, 58]. The formation of Pt^*δ*−^ in Pt–Ni_3_S_2_ was further confirmed by XANES measurements (Fig. 2e). The white-line peak, which can be observed at 11,566 eV in the XANES of Pt *L*-edge spectra, corresponds to an electron transition generated by the filled Pt 2*p*_3/2_ orbital. The white line of Pt–Ni_3_S_2_ in Fig. 2e comparison to Pt foil demonstrates the Pt 5*d* occupancy, which is consistent with presence of Pt^*δ*−^. These results indicate that electron transfer takes place from Ni_3_S_2_ nanocrystals to Pt nanocrystals. Instead, electron transfer takes place Pt nanoparticles to the carbon support in the commercial Pt/C [59–62]. The FT-EXAFS of Pt–Ni_3_S_2_ exhibits prominent peaks in the region 1.8–2.8 Å (Fig. 2f). The EXAFS of Pt–Ni_3_S_2_ displays the length of the Pt–Pt bond (~ 2.42 Å), which is approximately 6.2% shorter than that in Pt foil (~ 2.58 Å), demonstrating that the short-range ordered Pt–Pt bond is affected by Ni_3_S_2_ to make it form Pt–Pt(Ni) chemical bonding interactions at the Pt–Ni_3_S_2_ interface [34, 35, 63]. The Pt L_3_-edge EXAFS results also indicated the existence of Pt–S bond at ~ 1.92 Å, on which the powerful correlation between Pt and Ni_3_S_2_ is beneficial for anchoring Pt atoms to make Pt nanocrystals uniformly dispersed on the surface of Ni_3_S_2_ nanocrystals [64, 65]. Additionally, the WT-EXAFS analyses for Pt–Ni_3_S_2_, Ni_3_S_2_, Ni foil, Pt foil, and Ni(OH)_2_ are given in Figs. 2g–n and S4 for further investigating the coordination environment of Ni, S and Pt atoms. The WT-EXAFS spectra in Ni *K*-edge for Ni_3_S_2_ and Pt–Ni_3_S_2_ (Fig. 2g–l) show a maximum intensity at ~ 6.35 and ~ 5.70 Å^−1^, which further explain that the Ni–S bond length of Pt–Ni_3_S_2_ is shorter than the pristine Ni_3_S_2_ [25]. In addition, a WT intensity maximum (Fig. 2i–n) at about 9.8 Å^−1^ was attributed to the Pt–Pt scattering in Pt foil. In contrast, the intensity maximum of Pt–Ni_3_S_2_ is near 6.5 Å^−1^, assigned to the Pt–Pt(Ni) contribution [17, 27, 66]. All of these results inferred that the interface electronic interaction of Pt–Ni_3_S_2_ can lead to an asymmetrical charge distribution at Pt–Ni_3_S_2_ interface, in which the electrons are transferred from Ni_3_S_2_ nanocrystals to the Pt nanocrystals, resulting in the formation of high-valent Ni sites and negatively-charged Pt^*δ*−^ at the interface [62].

### Performance and Products Analysis for Electrocatalyst

**Fig. 3:**
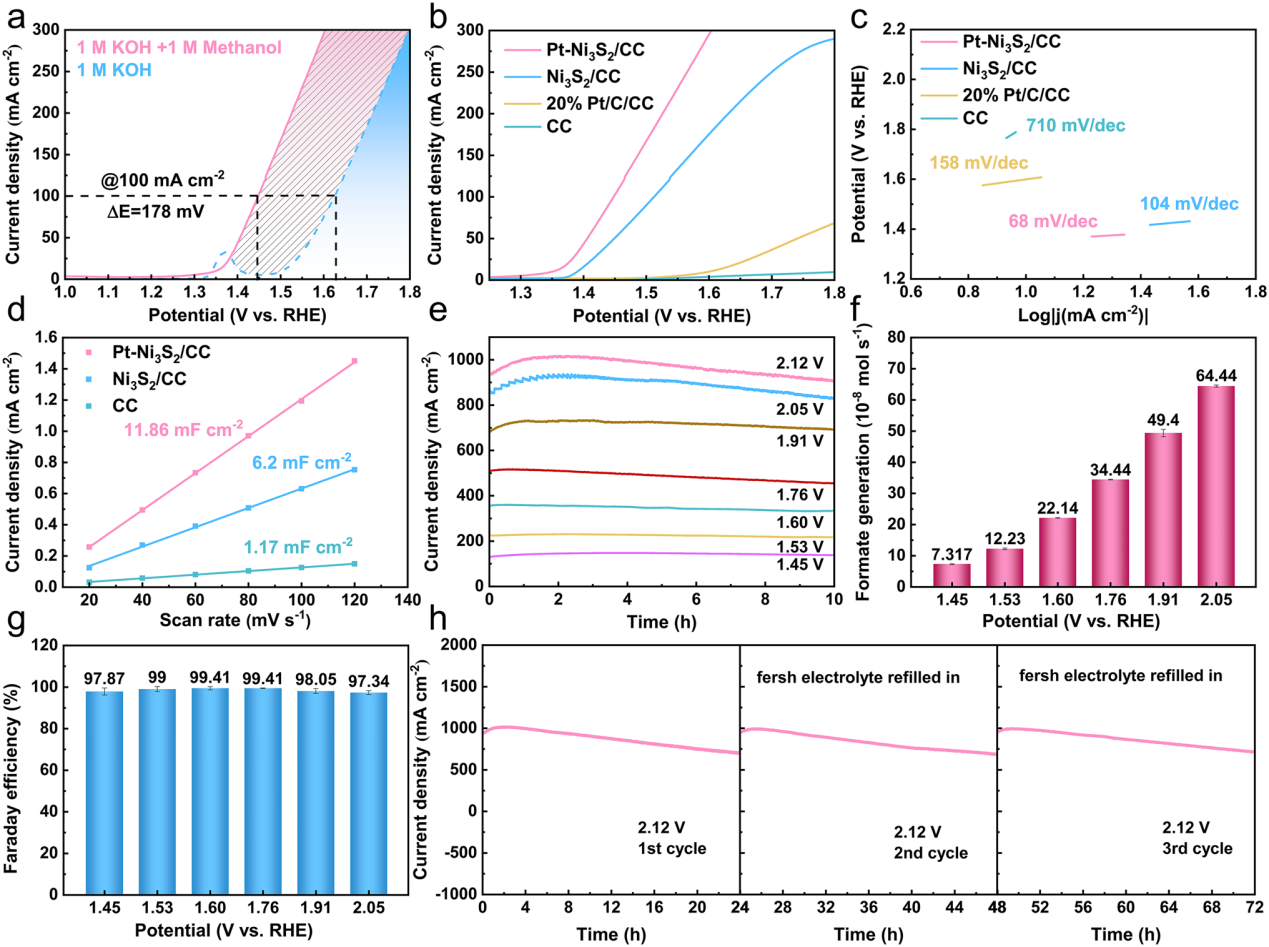
Electrocatalytic performance of all examined catalysts for methanol oxidation reactions (MOR). **a** LSV curves of Pt–Ni_3_S_2_/CC for MOR and OER; **b** LSV curves and **c** Tafel slope plots of Pt–Ni_3_S_2_/CC, Ni_3_S_2_/CC, 20% Pt/C/CC, CC; **d** calculated electrochemical double-layer capacitances (*C*_*dl*_) of Pt–Ni_3_S_2_/CC, Ni_3_S_2_/CC, CC; **e** chronoamperometry (*I-t*) curves at different potentials; **f** the formate generation rates at different potentials; **g** the faradaic efficiencies of formate obtained at different potentials; **h** cyclic stability studies at 2.12 V (vs. RHE)

The electrochemical performances and products analysis of Pt–Ni_3_S_2_ catalyst were examined in a three-electrode system. The reference electrodes used in the tests were calibrated according to the method in the literature (Fig. S5) [67]. The electrochemical behavior of the Pt–Ni_3_S_2_/CC catalyst was evaluated by LSV curves as shown in Fig. 3a. Apparently, the potential was reduced about 178 mV after the addition of methanol to KOH aqueous solution, suggesting that methanol molecules are more easily oxidized than the OH^−^. When the current density reaches at 200 mA cm^−2^, the potential was reduced about 188 mV after the addition of methanol to KOH aqueous solution. In typical LSV plots (Figs. 3b and S7), the Pt–Ni_3_S_2_/CC exhibits excellent performance compared with Ni_3_S_2_/CC, 20% Pt/C/CC, and CC without *iR*-compensation. Additionally, we found that the decreased oxidative potentials of Pt–Ni_3_S_2_/CC gradually increase after 50 mA cm^−2^ compared with Ni_3_S_2_/CC electrode (Fig. S10) [68]. For comparison, we also tested the catalytic activities toward OER by using Pt–Ni_3_S_2_/CC and other electrocatalysts under the same condition (Fig. S6). As shown in Fig. S6c, Pt–Ni_3_S_2_/CC demonstrated the largest current density at 1.6 V (vs. RHE) between MOR and OER. The better reaction kinetics of Pt–Ni_3_S_2_/CC for MOR was also confirmed with its lower Tafel slope of 68 mV mV dec^−1^ compared with Ni_3_S_2_/CC (104 mV dec^−1^) (Fig. 3c). Such results with *iR*-compensation are also presented in Fig. S9. Furthermore, the Nyquist plots (Fig. S11) show that Pt–Ni_3_S_2_/CC exhibits the lowest charge transfer resistance when compared with Ni_3_S_2_/CC and CC. We determined the double-layer capacitance of catalyst, which is inversely correlated with the electrochemical active surface area (ECSA), to better understand the causes of the high MOR activity. Analyses and calculations show the *C*_*dl*_ value of Pt–Ni_3_S_2_/CC (11.86 mF cm^−2^), superior to those of Ni_3_S_2_/CC (6.2 mF cm^−2^) and CC (1.17 mF cm^−2^), indicating that more active sites effective to MOR electrolysis exist in the heterojunction structure (Figs. 3d and S12). Due to the excellent dispersibility of Pt–Ni_3_S_2_ nano-heterojunctions, it is easy to form a large quantity of interfacial defects to expose more active sites, which should thus be the intrinsic reason for the highly activity of Pt–Ni_3_S_2_ electrocatalyst. Apart from the effect of active sites, intrinsic activity also plays a crucial role in optimizing overall MOR activity, which could be estimated by the turnover frequency (TOF). As shown in Fig. S8, when the potential of 1.475 V (vs. RHE) is applied, a TOF of 2 × 10^−2^ s^−1^ is recorded for the Pt–Ni_3_S_2_ catalyst, which is 1.43 and 4.95 times higher than that of the Ni_3_S_2_/CC, 20% Pt/C/CC, respectively, further confirming the outstanding intrinsic activity of the Pt–Ni_3_S_2_ catalyst in MOR catalysis. The working stability is also a crucial component when evaluating the various capabilities of the catalyst. In this work, the MOR stability of Pt–Ni_3_S_2_/CC was evaluated by *I–t* curves at different potentials. Figure 3e further displays the long-term stability of catalysts under different voltages, especially at high current density. Continuous electrolysis is carried out under different voltages to detect the value-added product at the anode, and Fig. 3f shows the generation of oxidized chemicals determined by ion chromatography (IC), signifying that the only available product is the value-added format via selective electrooxidation of methanol with Pt–Ni_3_S_2_/CC electrocatalyst at the anode. The amounts of formate were calculated by the standard calibration curve (Fig. S13), and the averaged generation rates of formate also increase with the increase of voltage (Fig. 3f), and the corresponding FEs almost reach 98% for the MOR at varied working potentials above (Figs. 3g and S14). All these further indicate that Pt–Ni_3_S_2_/CC electrocatalyst will not further oxidize methanol to environmental-unfriendly CO_2_, but oxidize methanol to formate products with higher commercial value. After three consecutive cycles of *I–t* curves tests, the catalytic activity of Pt–Ni_3_S_2_/CC can be recovered at approximately 1 A cm^−2^ by replacing fresh working electrolyte (Fig. 3h), implying the well reproducibility of MOR electrocatalysis. Furthermore, the main reason for the decrease of current densities in *I–t* curves is attributed to the pH change of electrolyte due to the consumption of the hydroxyl ions, which is elucidated by Eqs. S1–S3 in Supplementary Information. Such an excellent MOR performance of Pt–Ni_3_S_2_ also exceeds that of most reported catalysts (Table S4). All these results show that the Pt–Ni_3_S_2_ exhibit outstanding electrocatalytic activity.

The used Pt–Ni_3_S_2_ are further characterized by XPS and HRTEM after MOR stability tests by chronoamperometry (*I–t*) at 2.12 V (vs. RHE) with an initial current density of ~ 1000 mA cm^−2^ for 72 h. As shown in Fig. S15, the used Pt–Ni_3_S_2_ exhibits a positive shift in the binding energy of Ni 2*p*_1/2_ and Ni 2*p*_3/2_ of Ni^2+^ in Pt–Ni_3_S_2_ compared with the fresh one, accompanied with a little decrease of the shoulder peak at ~ 853 eV belonging to metallic Ni, indicating the increase of Ni atoms’ valence after such drastic methanol conversion by *I–t*. The global TEM image in Fig. S16a and HAADF-STEM image in Fig. S16b indicate that the heterojunction between Pt and Ni_3_S_2_ could be kept after such drastic electrocatalytic reaction, although the Ni_3_S_2_ nanoparticles are tightly aggregated with carbon black powders. The corresponding EDS elemental mapping images (Fig. S16c–f) of used Pt–Ni_3_S_2_ show good dispersion of elemental of Ni, S, C and Pt. In particular, elemental of Pt maintains excellent dispersibility after such drastic MOR process by chronoamperometry (*I–t*) at 2.12 V (vs. RHE) with an initial current density of ~ 1000 mA cm^−2^ for 72 h. The supplementary XPS and TEM results indicate that the hetero-structure of Pt–Ni_3_S_2_ is highly stable for electrocatalytic evolution at large current density, which further confirms its high potential for practical applications in environmental and energy fields.

### Theoretical Study and Mechanistic Insight

**Fig. 4:**
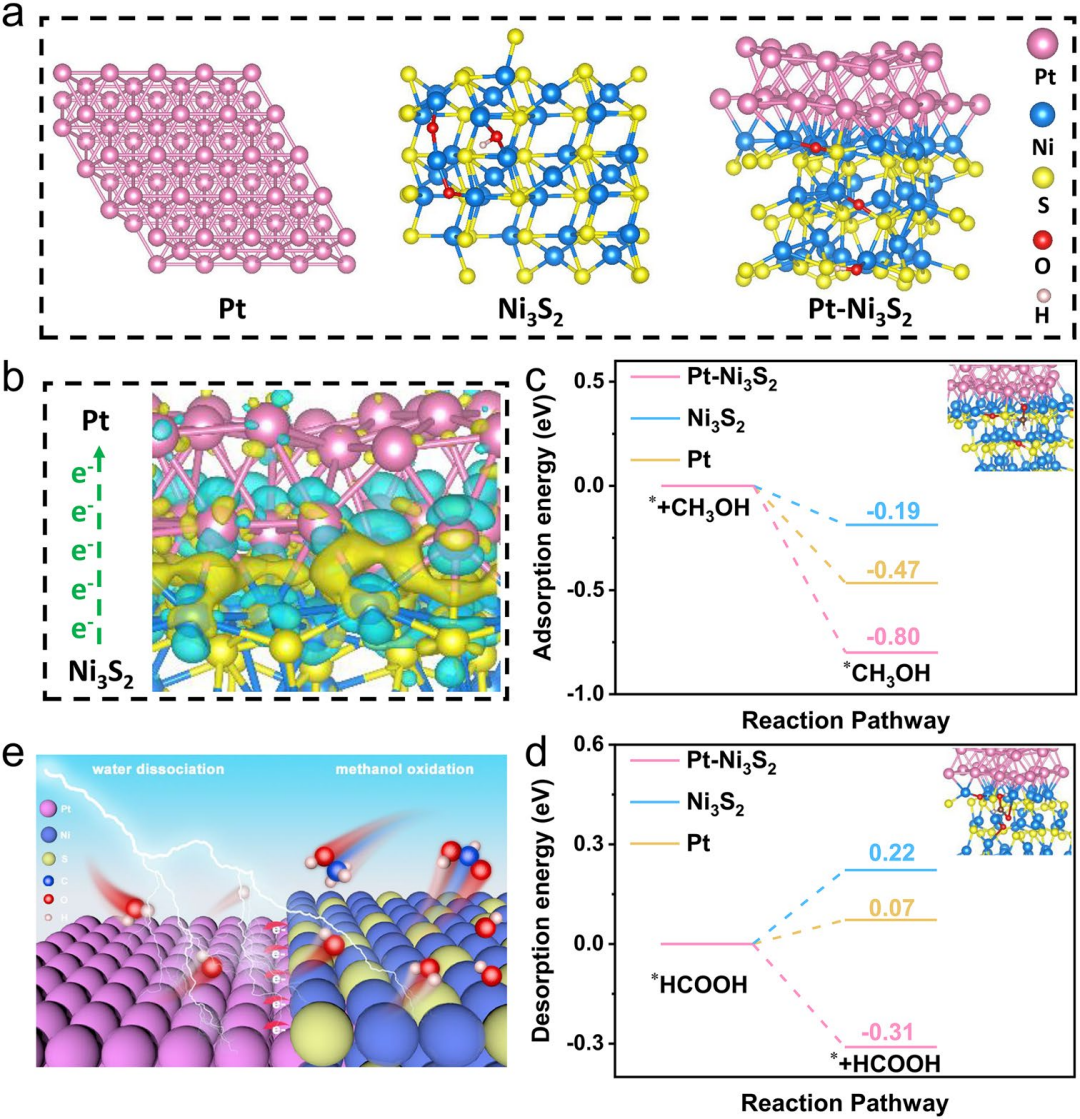
Density functional theory (DFT) computations. **a** Theoretical models of Pt foil, Ni_3_S_2_, and Pt–Ni_3_S_2_; **b** Charge density difference plot at the Pt–Ni_3_S_2_ interface; **c** the adsorption energy diagrams of CH_3_OH and **d** the desorption energy diagrams of HCOOH on the surfaces of Pt–Ni_3_S_2_, Ni_3_S_2_, and Pt foil; **e** Schematic illustration of MOR mechanisms for Pt–Ni_3_S_2_ electrocatalyst

To deeply understand the origin of the good performance of the Pt–Ni_3_S_2_ for MOR, DFT computations are further performed to gain insight into the interaction between Pt and Ni_3_S_2_ nanocrystals. The schematic models of Pt–Ni_3_S_2_, Ni_3_S_2_, and Pt foil catalyst are shown in Fig. 4a. These electronic changes were marked by the differential charge density of Pt–Ni_3_S_2_ interface, in which blue stands for electronic consumption state and yellow stands for electronic accumulation state (Fig. 4b). The charge density of Ni_3_S_2_ nanocrystals was weakened after introducing Pt nanocrystals, which laterally reflects the higher oxidation state of element Ni. Further, we calculated the charge distribution to quantitatively evaluate these charges. It can be clearly seen that the electrons at the interface are clustered on the Pt side.

These results indicate that the electronic interaction caused an electron aggregation effect on the Pt species and enhanced the oxidation state of Ni species, resulting in a significantly lower potential [69]. As shown in Fig. 1b, the onset potential of Pt–Ni_3_S_2_ is 1.31 V (vs. RHE) and its overpotential is 1.35 V (vs. RHE) at 10 mA cm^−2^, indicating that the electro-oxidation of adsorbed methanol is probably driven by the active Ni^*δ*+^ sites with higher valence (*δ* > 2, e.g., probably Ni–OOH), which is consistent with the reported literature about nickel-based electrocatalyst [15, 70]. As the potential gradually increases, methanol is decomposed to formic acid on the surface of the catalyst. The adsorption energy of CH_3_OH* and the desorption energy of HCOOH* is different due to the different electronic valence states of surface Ni on Pt–Ni_3_S_2_, Ni_3_S_2_, and Pt. Hence, their onset potentials and overpotentials are different accordingly. Further, the adsorption energy of CH_3_OH* and the desorption energy of HCOOH* on the surfaces of Pt–Ni_3_S_2_, Ni_3_S_2_, and Pt foil was calculated to analyze the adsorption/desorption performance of the products via DFT (Figs. 4c, d and S17–S19). The Pt–Ni_3_S_2_ shows stronger adsorption energy (− 0.80 eV) for CH_3_OH* than Pt (− 0.47 eV) and Ni_3_S_2_ (− 0.19 eV), which indicates that Pt–Ni_3_S_2_ has a strong methanol adsorption capacity. It is beneficial for the initial adsorption and activation of CH_3_OH [71]. Figure 4d shows that the energy span for *HCOOH → * + HCOOH is − 0.31 eV on Pt–Ni_3_S_2_ surface, which is lower than those on Pt foil (0.22 eV) and Ni_3_S_2_ (0.07 eV), indicating that this step is thermodynamically easier to occur on Pt–Ni_3_S_2_ surface where Pt acts as the catalyst promoter. Therefore, the strong chemical interaction between Pt and Ni_3_S_2_ can modulate the electronic structure of Pt–Ni_3_S_2_, which further facilitates the formation of high-valent Ni and enhances the adsorption energies of intermediates for MOR, resulting in MOR activity [72]. Based on the aforementioned analysis, an active center mechanism for Pt–Ni_3_S_2_ to catalyze MOR is discussed (Fig. 4e). The methanol molecule, initially adsorbed on the catalyst surface via forming Ni^3+^–O bond, will undergo successive de-protonation and C–H bond cleavage steps with the assistance of Pt site. Finally, the intermediate at the Ni site is formed and easily converted into HCOO^−^ due to the high concentrations of OH^−^ in the electrolyte [15, 23, 69].

**Fig. 5:**
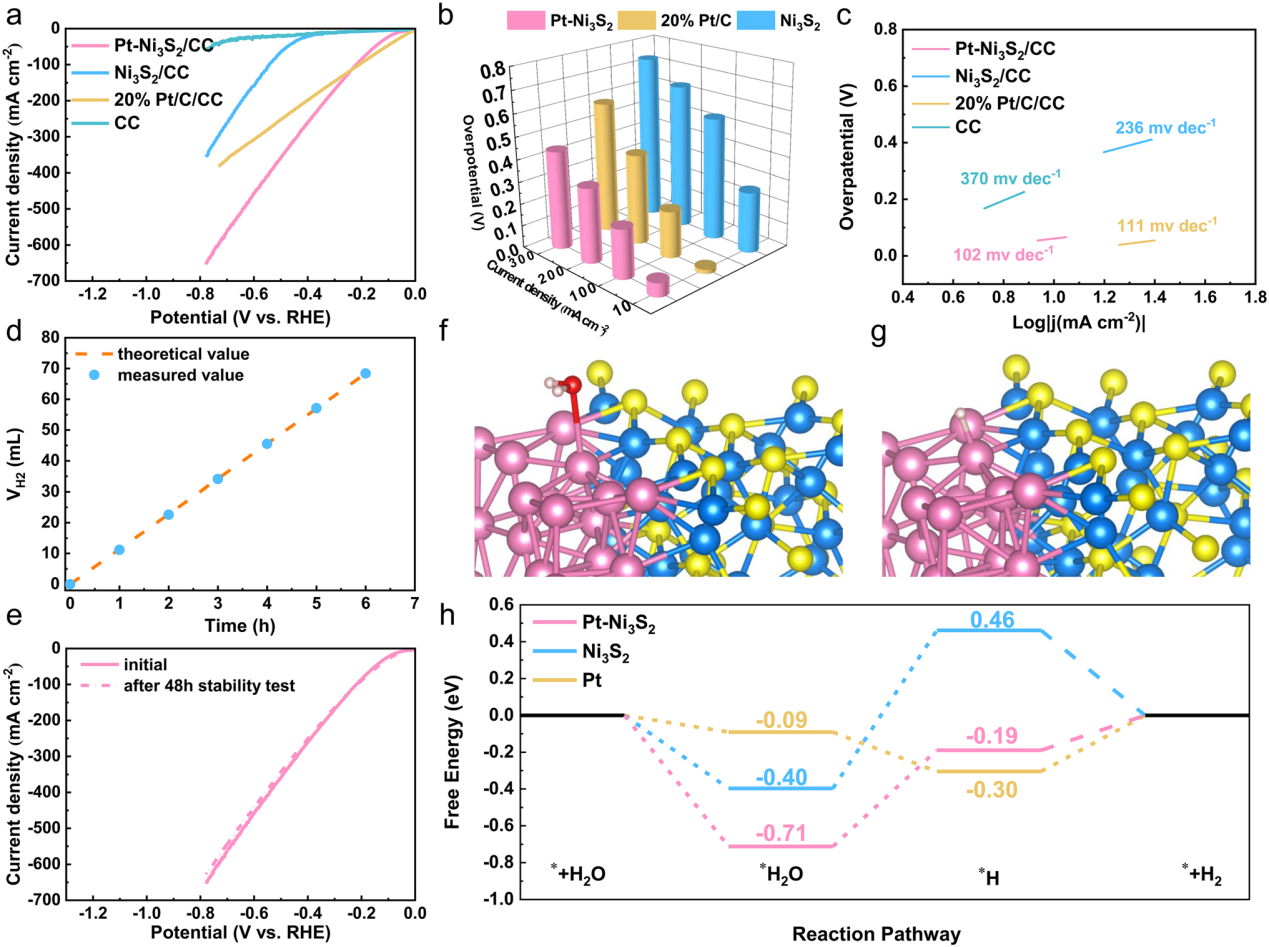
Electrocatalytic performance of all examined catalysts for HER without *iR*-compensation. **a** LSV curves; **b** bar diagram representing the overpotentials at different current densities; **c** Tafel curves; **d** the calculated theoretical values and the measured H_2_ amount; **e** the HER stability test after 48 h by the LSV curves; **f** the adsorbed H_2_O on Pt–Ni_3_S_2_ model structures; **g** the adsorbed H on Pt–Ni_3_S_2_ model structures; **h** the H_2_O and H adsorbing free energy diagrams on catalyst surfaces

The electrocatalytic HER performance of Pt–Ni_3_S_2_ and Ni_3_S_2_ catalysts was studied using a standard three-electrode system at room temperature. Meanwhile, Commercial 20% Pt/C was used as a comparative sample to compare the HER activity of Pt–Ni_3_S_2_ and Ni_3_S_2_. The LSV curves (Fig. 5a) show that Pt–Ni_3_S_2_ exhibited the higher HER activity with a lower *η*_10_ of 61 mV (vs. RHE) than that of pure Ni_3_S_2_ (270 mV) without *iR*-compensation. The data with *iR*-compensation (Fig. S22) also confirms the performance tendency of these three kinds of electrocatalysts. Interestingly, Pt–Ni_3_S_2_ gradually surpassed the 20% Pt/C at a high current density region. That is, Pt–Ni_3_S_2_ requires an ultralow working potential of 440 mV (587 mV for 20% Pt/C) to achieve 300 mA cm^−2^. As histogram (Fig. 5b) also visually shows its advantages over other catalysts. Such an excellent HER performance of Pt–Ni_3_S_2_ also exceeds that of mostly reported catalysts (Table S5). Mass activity is also an important parameter for evaluating the electrocatalytic activity. As given in Fig. S20, Pt–Ni_3_S_2_/CC exhibits much higher mass activity than 20% Pt/C/CC at higher overpotential. The HER kinetics and mechanism are uncovered by Tafel slopes in Fig. 5c, and the Pt–Ni_3_S_2_ with 102 mV dec^−1^ is lower than 20% Pt/C (111 mV dec^−1^) and Ni_3_S_2_ (236 mV dec^−1^). This result is further validated by the lowest *R*_ct_ of 2.4 Ω in the EIS spectra in Fig. S23. Further, ECSA normalized polarization curve (Fig. S24) shows the *C*_*dl*_ value of Pt–Ni_3_S_2_/CC is 18.75 mF cm^−2^, which is strongly superior to those of Ni_3_S_2_/CC (11.64 mF cm^−2^) and CC (1.86 mF cm^−2^), reflecting that the Pt–Ni_3_S_2_ has a higher amount of catalytic active sites. In addition, the Pt–Ni_3_S_2_ shows nearly 100% Faradaic efficiency by comparing theoretical value and measured value (Figs. 5d and S25). The cycling stability of Pt–Ni_3_S_2_ catalyst has been further verified by LSV scanning, in which the polarization curves measured by chronoamperometry after 48 h almost overlaps with the initial polarization curve, signifying the strong HER stability (Fig. 5e). The used Pt–Ni_3_S_2_ after HER stability tests are further characterized by XPS (Fig. S26) and HRTEM (Fig. S27), in which the results are similar to the those after MOR stability tests, indicating that the used Pt–Ni_3_S_2_ can operate stably for 24 h at a high current density of 100 mA cm^−2^ with remained excellent dispersibility of Pt atoms. TOF is another important figure of merit used to reveal the intrinsic electrocatalytic activity. As shown in Fig. S21, the Pt–Ni_3_S_2_/CC show much higher TOF values over the whole potential ranges than the other catalysts for HER. The TOF values of Pt–Ni_3_S_2_/CC were 1.44 and 2.88 s^−1^ at the overpotential of 50 and 100 mV in 1.0 mol L^−1^ KOH, respectively, which are all superior to other catalysts. Moreover, DFT calculations are applied to provide the mechanistic understandings for the high activity of Pt–Ni_3_S_2_ toward HER. Normally, the key steps affecting the reaction rate for alkaline HER include water adsorption and hydrogen desorption. Hence, the Gibbs free energy for hydrogen adsorption (Δ*G*_H*_) on electrocatalysts are calculated in this study. Figures 5f–g and S28–S30 show the binding models of H_2_O molecule and H atom at the active sites of Pt on Pt–Ni_3_S_2_. According to DFT simulation (Fig. 5h), it should be a rate determining step from the dissociation of *H_2_O to the formation of *H on Pt–Ni_3_S_2_, since a larger energy gap exists between *H_2_O (− 0.71 eV) and *H (− 0.19 eV). Hence, the HER performance on 20% Pt/C is better than that on Pt–Ni_3_S_2_ at lower overpotential, which is indicated by the LSV results in Fig. 5a. However, the current density of HER on Pt–Ni_3_S_2_ (Fig. 5a) is obviously larger than that on 20% Pt/C at higher overpotential, probably because the energy barrier between *H_2_O and *H is overcome on such condition [62, 65, 73]. Furthermore, the Δ*G*_H*_ is calculated to determine the activity of catalysts during the adsorption of hydrogen atoms. According to the DFT calculation results, the ∆*G*_H*_ of Pt–Ni_3_S_2_ (− 0.19 eV) is closer to 0 eV compared with Ni_3_S_2_ (0.46 eV) and Pt foil (− 0.30 eV), demonstrating a more favorable H* desorption (Fig. 5h), probably owing to the fact the electron-enriched Pt atoms at Pt–Ni_3_S_2_ are not easy to be oxidized and can efficiently adsorb hydrogen species to obtain more moderate H binding energy. This could dramatically facilitate the conversion of intermediates and desorption of H_2_, thereby improving the performance of the catalyst for long-term stable alkaline electrolysis of aquatic hydrogen [6, 74, 75].

### Overall Methanol Splitting Performance

**Fig. 6:**
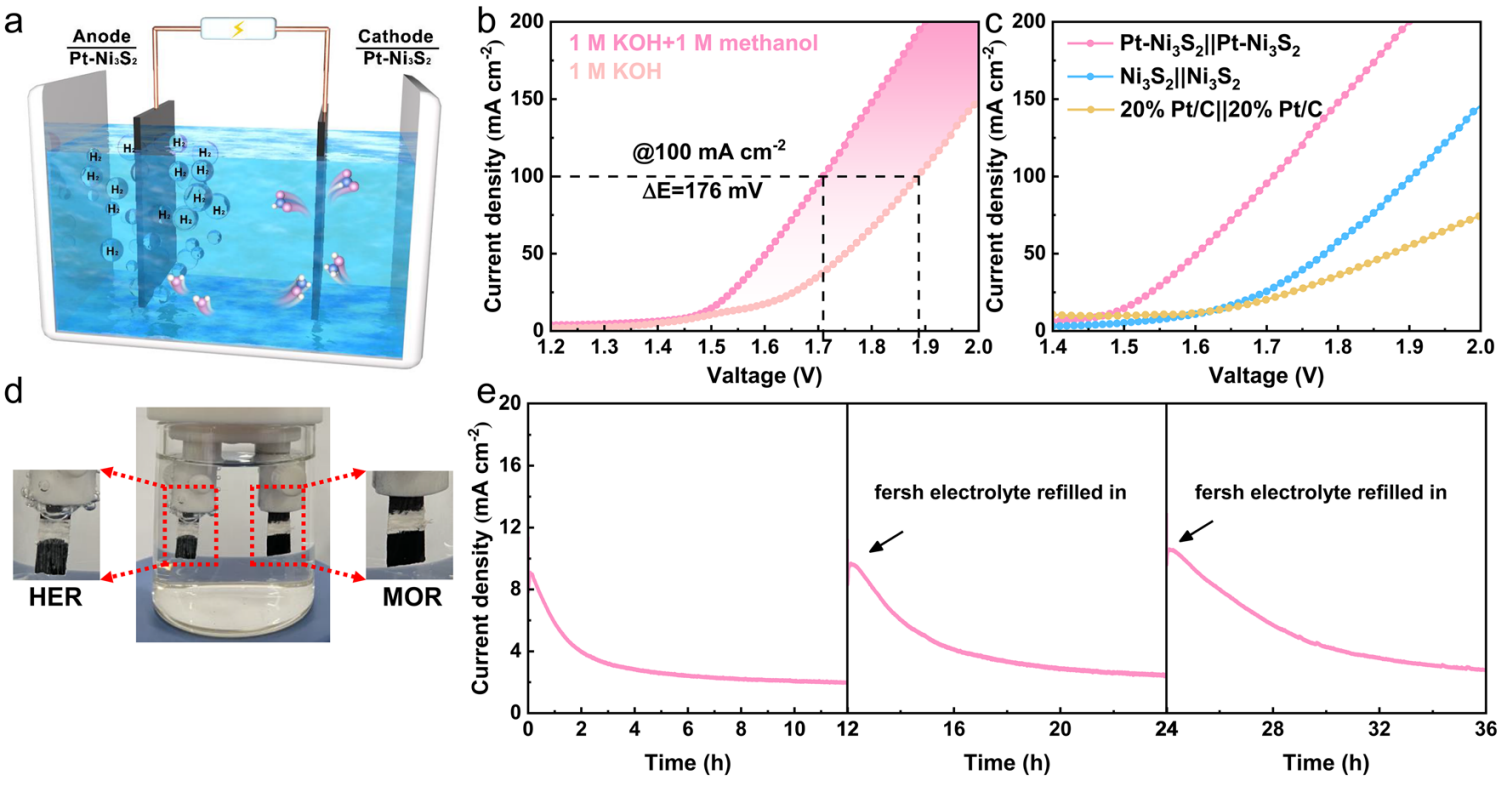
Schematic illustration of two-electrode system. **a** Illustration of the assembled electrocatalytic system of methanol–water electrolyzer; **b** Comparison of the overall water splitting and methanol–water co-electrolysis by using Pt–Ni_3_S_2_; **c** Comparing the co-electrolytic performances with other electrocatalysts; **d** the photo of the two-electrode configuration during operation; **e** the stability test of Pt–Ni_3_S_2_

Considering the excellent activity and stability of Pt–Ni_3_S_2_ for HER and MOR, we believe that Pt–Ni_3_S_2_ can be an excellent bi-functional catalyst in the decomposition of methanol- water. Hence, a two-electrode cell using Pt–Ni_3_S_2_ electrocatalysts as both electrodes were established in 1.0 mol L^−1^ KOH with the presence of 1.0 mol L^−1^ methanol (Fig. 6a). As indicated in Fig. 6b, the cell voltage for Pt–Ni_3_S_2_ in the methanol–water electrolyzer is merely 1.71 V to drive a current density of 100 mA cm^−2^, which is 176 mV lower than that in the overall water electrolyzer, demonstrating more efficient hydrogen production with the assistance of methanol selective upgrading. At the same time, the electrolytic performance is also compared with different dual-electrodes systems including Ni_3_S_2_||Ni_3_S_2_, and 20% Pt/C||20% Pt/C (Fig. 6c). The cell voltages reaching 50 mA cm^−2^ for the latter two systems are 1.78 V and 1.82 V, respectively, which are all higher than the Pt–Ni_3_S_2_ || Pt–Ni_3_S_2_ system (1.60 V). As the current density increases, the voltage difference between Pt–Ni_3_S_2_ and other comparison samples will be further increased. Furthermore, the 36 h’ chronoamperometry (*I–t*) measurement has been implemented by cyclically refilling the fresh electrolyte (1.0 mol L^−1^ KOH + 1.0 mol L^−1^ methanol). The *I–t* curve in Fig. 6e indicates that the Pt–Ni_3_S_2_ shows the advantages of excellent long-term stability and cyclic reusability for methanol–water co-electrolysis, demonstrating its good prospect of practical applications.

## Conclusion

In summary, the dual-monodispersed Pt–Ni_3_S_2_ heterojunction nanocrystals (“*DMD* Pt–Ni_3_S_2_ *HNCs*”) with good dispersion rich interface defects are constructed as highly active electrocatalysts by anchoring platinum on Ni_3_S_2_ nanocrystals through the injection method. The “*DMD* Pt–Ni_3_S_2_ *HNCs*” with fully exposed active sites exhibit excellent bi-functional activity and stability towards HER and MOR, which require only 1.45 V (vs. RHE) to achieve 100 mA cm^−2^ for MOR and a low *η*_10_ of 61 mV for HER, respectively. Coupled with XAFS and DFT calculations, it shows that the electronic interactions at the interface of dual-monodispersed heterojunctions result in an asymmetrical charge distribution at Pt–Ni_3_S_2_ interface. On the one hand, the positive charge-enriched Ni_3_S_2_ area could promote and stabilize high-valent Ni sites to effectively optimize and facilitate the oxidation process of reaction intermediates, resulting in high electrocatalytic activity and selectivity for MOR. On the other hand, the negative charge-enriched Pt side is responsible for optimizing the H* conversion and H_2_ desorption to accelerate water dissociation, improving performance for HER. Further, an alkaline electrolysis cell of Pt–Ni_3_S_2_||Pt–Ni_3_S_2_ exhibits outstanding activity, which requires a low cell voltage of 1.60 V to drive a current density of 50 mA cm^−2^. The construction of heterostructured interfaces to modulate surface charge distribution provides a new pathway for superior bi-functional electrocatalysts to achieve the concurrent production of value-added formate and hydrogen.

## Supplementary Information

Below is the link to the electronic supplementary material.

Supplementary file1 (PDF 2840 KB)
