# Supplementary material for: Interfacial Electronic Modulation of Dual-Monodispersed Pt–Ni3S2 as Efficacious Bi-Functional Electrocatalysts for Concurrent H2 Evolution and Methanol Selective Oxidation
Source: Nanomicro Lett. 2024 Jan 11;16:80. doi: 10.1007/s40820-023-01282-4 (PMC10784266; doi:10.1007/s40820-023-01282-4)
Supplement: Supplementary file 1 — Supplementary file1 (PDF 2840 KB) [file 40820_2023_1282_MOESM1_ESM.pdf]

Supporting Information for

Interfacial Electronic Modulation of Dual-Monodispersed Pt-Ni_3S_2 as Efficacious Bi-functional Electrocatalysts for Concurrent H_2 Evolution and Methanol Selective Oxidation

Qianqian Zhao[1], Bin Zhao[1,*], Xin Long[1], Renfei Feng[2], Mohsen Shakouri[2], Alisa Paterson[2], Qunfeng Xiao[2], Yu Zhang[3], Xian-Zhu Fu[1], Jing-Li Luo[1,*]

[1] Shenzhen Key Laboratory of Energy Electrocatalytic Materials, Shenzhen Key Laboratory of Polymer Science and Technology, Guangdong Research Center for Interfacial Engineering of Functional Materials, College of Materials Science and Engineering, Shenzhen University, Shenzhen 518060, P. R. China

[2] Canadian Light Source Inc., Saskatoon, S7N 0X4, Saskatchewan, Canada

[3] Instrumental Analysis Center of Shenzhen University (Lihu Campus), Shenzhen University, Shenzhen, Guangdong 518055, P. R. China

*Corresponding authors. E-mail: bin.zhao@szu.edu.cn (Bin Zhao), jll@szu.edu.cn (Jing-Li Luo)

S1 Method

S1.1 Chemicals

All the reagents were used as received without further purification, including nickel (II) acetylacetonate (Ni(acac)$_2$; 96%), Platinum(II) acetylacetonate (Pt(acac)$_2$; 98%), diphenyl disulfide (DPDS; 96%), oleylamine (OLA; 90%) and Nafion solution (~5% in a mixture of lower aliphatic alcohols and water) were purchased from Sigma-Aldrich. KOH (GR, 95%), Toluene (AR, 99.5%), methanol (AR, 99.5%), and isopropanol (AR, 99.5%) were obtained from Sinopharm Chemical Reagent Co. Ltd. (Shanghai, China) and commercial 20% Pt/C obtained from Aladdin Chemical Reagent Co. Ltd. (Shanghai, China). All chemicals were used as received without any further purification.

S1.2 Synthesis of Ni_3S_2 catalyst

The one-pot solution-based syntheses were performed using a standard *Schlenk* vacuum line technique under argon atmosphere. In a standard synthesis, 2 mmol (0.5138 g) Ni(acac)$_2$ and OLA (20 mL) were fully dissolved in a round-bottom *Schlenk* flask (100 mL) at room temperature. The flask was degassed under vacuum at 80 °C for 0.5 h to remove oxygen and other low-boiling-point organic solventsin oil bath. Subsequently, the reaction was programmed to be 220 °C with a ramp of 5 °C min^{-1} after backfilling with Ar in oil bath. At the same time, 1 mmol (0.2184 g) DPDS and 3 mL OLA, 0.1 mmol (0.04 g) Pt(acac)$_2$ and 1 mL OLA was separately mixed in a glass vial, then preheated to 80 °C on a hot plate to form a clear solution. When the flask reaches 220 °C, the diphenyl disulfide solution was injected into the metal solution by syringe. After injection, the temperature drops to 210-215 °C and the reaction was allowed to maintain at 215 °C for 10 minutes with continuous stirring. After stopping the reaction, the flask was taken out of the oil bath and allowed to naturally cool to room

temperature. The product was dissolved in toluene and the solution was centrifuged at 12000 rpm during 10 minutes for nanoparticles separation. Finally, the as-synthesized Ni_3S_2 nanocrystals were thoroughly purified by multiple precipitation and re-dispersion steps using toluene and isopropanol.

1.3 Preparation of Working Electrode

The Pt-Ni_3S_2 catalyst modified carbon cloth is employed as both cathode and anode electrodes. Prior to the modification, carbon cloth (CC) was cut into 0.5 cm × 1.5 cm rectangular pieces, and then they were rinsed with water and ethanol thoroughly under sonication to remove residual organic species. For electrode modification, we use fresh suspensions to prepare ink by the following steps: the toluene suspension (1 mL) was separately dissolved in isopropanol and ethanol, and the solution was centrifuged at 10000 rpm during 5 minutes to precipitate metal complexes. Then the as-synthesized Pt-Ni_3S_2 catalyst and 5 mg carbon black was finally dispersed in ethanol (200 µL), and 25 µL 5 wt % Nafion solution. The mixed solution was followed by ultrasonication for 30 min to obtain a homogeneous catalyst suspension. Then 25 µL catalyst ink was dropped on the carbon cloth (loading area 0.5 cm × 0.5 cm) yielding a mass loading of 1.5 mg cm^{-2}. The catalyst modified electrode was dried at 60 °C before electrochemical measurements. At least three identical electrodes for each electrocatalysts were made for the repeatability test.

S1.4 Reference Electrode (RE) Calibration

We used Ag/AgCl as the reference electrode for all measurements. The calibration was performed in the high purity hydrogen saturated electrolyte with a Pt foil as the working electrode and counter electrode. CV were run at a scan rate of 1 mV s^{-1} in 1 M KOH with/without 1 M methanol, and the average of the two potentials at which the current crossed zero was taken to be the thermodynamic potential for the hydrogen electrode reactions. The calibrated potentials measured against Ag/AgCl with RE calibration was convert to the reversible hydrogen electrode was calculated as follow: E (RHE) = E (Ag/AgCl) + 1.0205 V (Fig. S5)[1, 2].

S1.5 Catalyst Characterization

The Pt-Ni_3S_2 catalyst were characterized by field emission transmission electron microscopy (FETEM) under the acceleration voltage of 200 kV and equipped with a EMSIS Xarosa CCD camera and Oxford INCA (Aztec) EDS detector at 20 kV with the potential of performing elemental analysis on the mode of dark field. To prepare the TEM specimens, one drop of the suspension was placed on a carbon film supported molybdenum grid and allowed to dry in air before the specimens were transferred into the microscope.

Crystallographic and purity information on Pt-Ni_3S_2 catalyst were obtained using powder XRD. To analyze these materials, the as-synthesized samples (dispersed in isopropanol) after centrifugation were later air-dried upon deposition onto glass slides. Diffraction patterns of these materials were collected using a powder diffractometer (RIGAKU Smartlab) operating in the reflection mode equipped with a Ni filter and a Cu Kα radiation (λ = 1.5406 Å) source with the accelerating voltage of 40 kV and the applied current of 200 mA. The scanning is performed at 10 ° min^{-1} from 10 ~ 80 ° with a step size of 0.02 °.

X-ray photoelectron spectroscopy (XPS) measurements were performed on a Thermo Scientific ESCALAB 250Xi spectrometer employing a monochromatic Al Kα X-ray source (hν = 1486.8 eV) and 500 μm test spot area under 15 kV test tube voltage, 10 mA tube current, and 2×10^{-9} mbar room floor vacuum. The takeoff angle for the collection of photoelectrons was 90 ° from the surface normal. Survey spectra were recorded on all samples followed by high resolution XPS spectra for Ni 2p, S 2p, Pt 2p and O 1s spectral regions. All the peaks were calibrated with C 1s spectrum at binding energy of 284.8 eV.

Ni *K*-edge X-ray absorption fine structure (XAFS) spectra of the Pt-Ni_3S_2 catalyst were recorded by synchrotron radiation light source at the VESPERS beamline of the Canadian Light Source (CLS) with medium energy range of 6-30 keV. The catalysts were dispersed on Kapton (polyimide) tape for XAFS measurements using a four element vortex detector in the transmission mode at room temperature. The Si (111) crystal monochromator was utilized to acquire the scan for X-ray spectra, and the data was collected in the total electron yield (TEY) mode by measuring the sample drain current. The XAFS spectra include X-ray absorption near edge structure (XANES) and extended X-ray absorption fine structure (EXAFS). Energy calibration was performed with a standard nickel foil by shifting all spectra to a glitch in the incident intensity. Each spectrum was mathematically processed by a standard normalization method to exclude the influence of sample thickness, absorber concentration, detector, and amplifier settings. The k3-weighted Fourier transformation of EXAFS (FT-EXAFS) and the k2-weighted Wavelet transforms of the EXAFS (WT-EXAFS) spectra were made to obtain the information on local electronic structures and radial distribution environment of Ni atoms in Pt-Ni_3S_2 catalyst.

1.6 Identification and Quantification of Formate Product

The identification and quantification of formate product are conducted by Ion Chromatography (IC) and determined by calibration curve. A small amount of electrolyte was taken by syringe after anode-cathode interchangeable electrocatalysis under ISTEP, and the electrolyte was properly diluted and consequently analyzed by Ion Chromatography (IC) to detect the formate generation. The Ion Chromatography (IC) was carried out on a CIC-D120 ion chromatograph (Shenghan Chromatography Technology Co., Ltd, Qingdao, China) equipped with an SH-AC-3 type anionic column. An aqueous solution containing sodium carbonate (2.4 mmol L^{-1}) and sodium bicarbonate (6.0 mmol L^{-1}) is employed as the eluent. The measurement is conducted at a constant temperature of 35 °C with a flow rate of 1.0 mL min^{-1}. At least three accurate trace curves were collected for statistical analysis.

The identification and quantification of the formate products were determined by calibration curve by applying standard formate solutions with known concentrations of commercially purchased pure sodium formate (chromatographic pure) (Fig. S13a).

The Faradaic efficiency (FE) of formate generation was calculated using the following equation:

$$\text{FE(formate, \%)} = \frac{\text{mole of formed formate}}{\text{total charge passed / } (4 \times F)} \times 100\%$$

$$\mathrm{FE(formate,\%)} = \frac{4 \times 96485(\frac{\mathrm{C}}{mol}) \times \omega_t(\frac{mg}{\mathrm{L}}) \times \mathrm{V(L)} \times 10^{-3}(\frac{\mathrm{g}}{mg})}{M_{formate}(\frac{\mathrm{g}}{mol}) \times \int_0^t I(A)dt} \times 100\%$$

where

F is the Faraday constant (96485 C mol^{-1}).

ω_t(mg L^{-1}) is the concentration of formed formate in the electrolyte, namely, the IC data (ppm). The unit of ppm here is mass(formate)/volume(solution).

V(L) is the total volume of the electrolyte.

$M_{formate}$(g mol^{-1}) is the molecular weight of formate ($HCOO^-$) equal to 45.02 g mol^{-1}.

I(A) is the current recorded by the electrochemical workstation in the ISTEP mode.

S1.7 Identification and Quantification of Gas Products

The generated H_2 from the electrolyzer were determined by gas chromatography (5977B MSD, Agilent Technologies) with a thermal conductivity detector (TCD). Argon (purity: 99.999%) was used as a carrier gas with a constant flow rate of 20 mL min^{-1}. A stable flow rate of mixed gas (the gaseous products and the carrier gas) was achieved after a period of electrocatalytic reaction, which gave the accurate GC traces for measurements. The GC sampling was conducted at 1, 2, 3, 4, 5 and 6 hours of electrocatalytic reaction. The identification and quantification of the H_2 products were determined by calibration curve by applying commercial standard H_2 gas with known concentrations.

The generation rate (mol s^{-1}) of H_2 was calculated using the following equation:

$$n_v(\mathrm{H_2}, mol/s) = \frac{\mathrm{V}vp_0}{\mathrm{RT_0}}$$

$$n_v(\mathrm{H_2}, mol/s) = \frac{\mathrm{V}(\frac{m^3}{s}) \times v(vol\ ratio) \times 1.01 \times 10^5(\frac{\mathrm{N}}{m^2})}{8.314(\frac{\mathrm{N}\ m}{mol\ \mathrm{K}}) \times 298.15(\mathrm{K})}$$

where

v(vol ratio) is the volume concentration of H_2 in the exhaust gas from the electrolyzer, namely, the GC data (volume ppm). The unit of ppm here is volume(H_2)/volume(total).

V(m^3 s^{-1}) is the gas flow rate measured by a flow meter at room temperature and under ambient pressure.

The Faradaic efficiency (FE) of H_2 production was calculated using the following equation:

$$\mathrm{FE(H_2,\%)} = \frac{\text{mole of formed } \mathrm{H_2}}{\text{total charge passed } / \ (2 \times F)} \times 100\%$$

$$\mathrm{FE(H_2,\%)} = \frac{2\mathrm{FV}vp_0}{\mathrm{RT_0}I} \times 100\%$$

$$FE(H_2,\%) = \frac{2 \times 96485(\frac{C}{mol}) \times V(\frac{m^3}{s}) \times v(vol\ ratio) \times 1.01 \times 10^5(\frac{N}{m^2})}{8.314(\frac{N\ m}{mol\ K}) \times 298.15(K) \times I(A)} \times 100\%$$

where

F is the Faraday constant (96485 C mol^{-1}).

v(vol ratio) is the volume concentration of H_2 in the exhaust gas from the electrolyzer, namely, the GC data (volume ppm). The unit of ppm here is volume(H_2)/volume(total).

V(m^3 s^{-1}) is the gas flow rate measured by a flow meter at room temperature and under ambient pressure.

I(A) is the current recorded by the electrochemical workstation in the ISTEP mode.

S2 Supplementary Figures

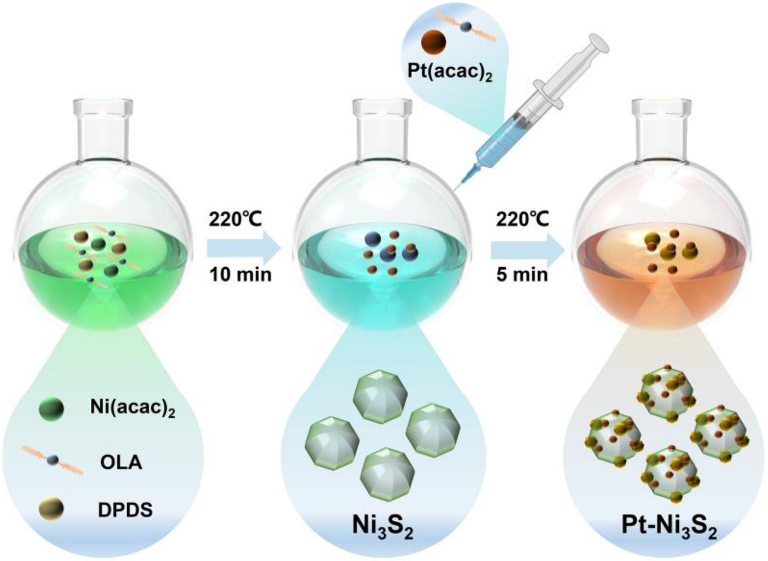

Scheme S1 Diagram of the synthesis of Ni_3S_2 and Pt-Ni_3S_2

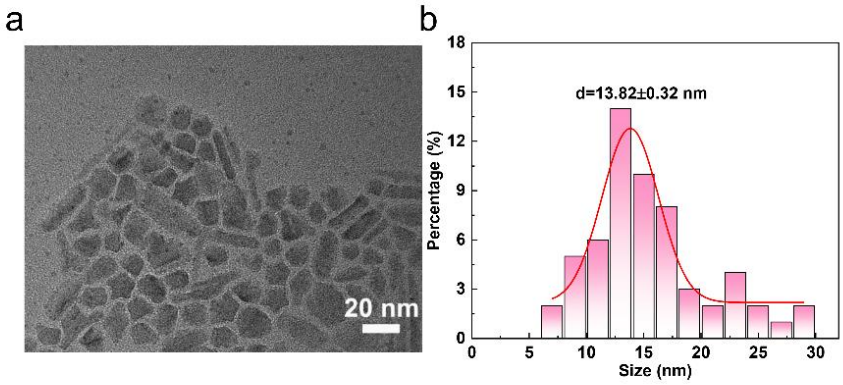

Fig. S1 TEM image and size distribution plot clearly shows a monodispersed system with a particle size of 13.82 ± 0.32 nm of Ni_3S_2 nanocrystals

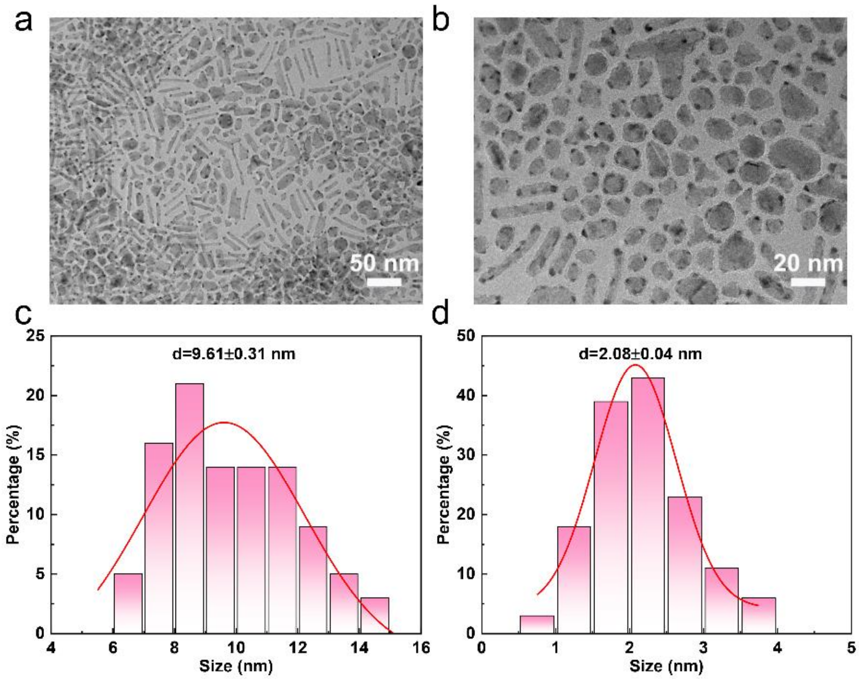

Fig. S2 TEM image and size distribution plot clearly shows a dual-monodispersed system with a particle size of 9.61 ± 0.31 nm of Pt-Ni_3S_2 nanocrystals, in which, monodisperse Pt particles are only 2.08 ± 0.04 nm in size

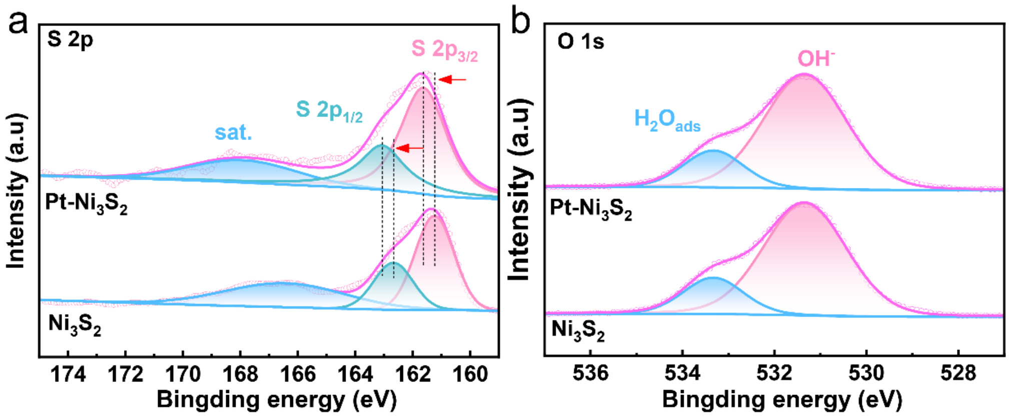

Fig. S3 XPS of (**a**) S 2p and (**b**) O 1s of Pt-Ni_3S_2 and Ni_3S_2

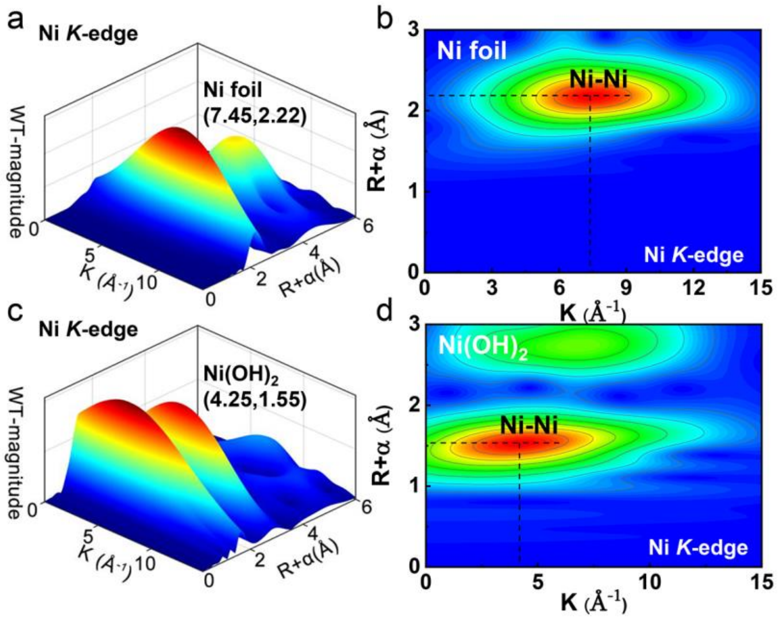

Fig. S4 Wavelet transformed k2-weighted EXAFS spectra (WT-EXAFS) and the corresponding 2D graphics of WT-EXAFS spectra about Ni for (**a-b**) Ni foil, (**c-d**) $Ni(OH)_2$

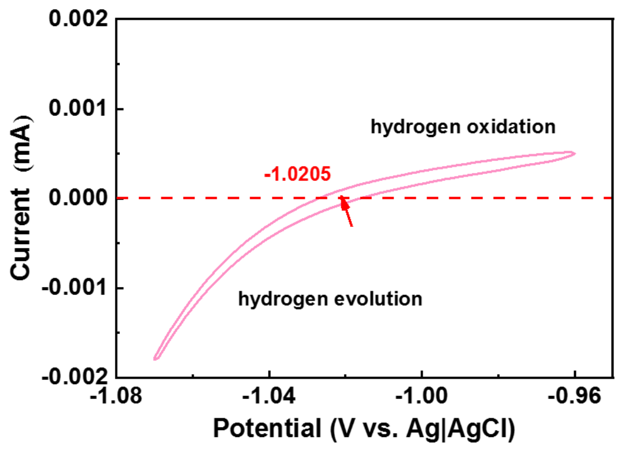

Fig. S5 The calibration curve of Ag/AgCl in 1 mol L^{-1} KOH with 1 mol L^{-1} methanol

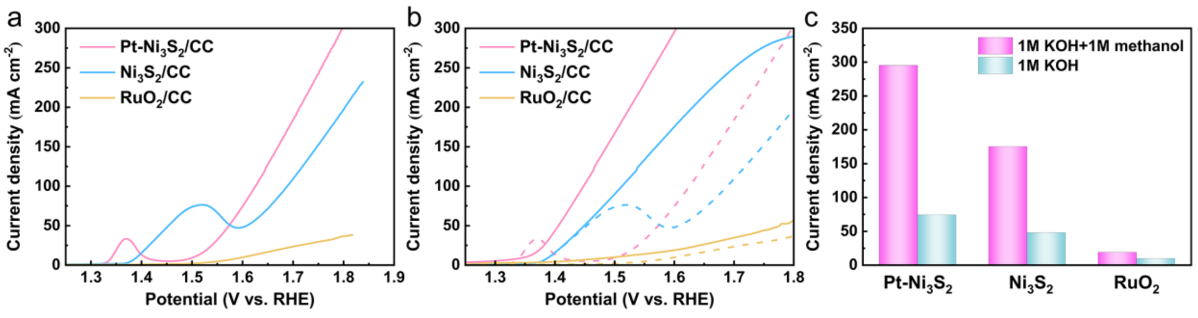

Fig. S6 (**a**) LSV curves of Pt-Ni_3S_2/CC, Ni_3S_2/CC, RuO_2/CC in 1.0 mol L^{-1} KOH without *iR*-compensation; (**b**) LSV curves of Pt-Ni_3S_2/CC, Ni_3S_2/CC, RuO_2/CC in 1.0 mol L^{-1} KOH electrolyte with (solid lines)/without (dot-dash lines) the addition of 1.0 mol L^{-1} methanol without *iR*-compensation. (**c**) The current density performance gap between MOR and OER at 1.6 V (vs. RHE)

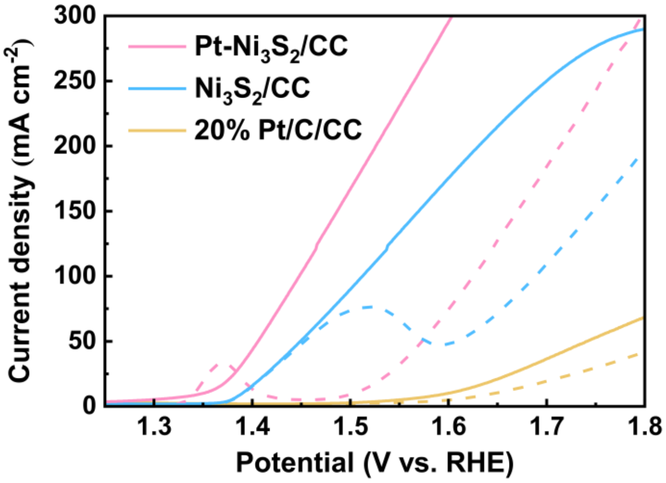

Fig. S7 LSV curves of Pt-Ni_3S_2/CC, Ni_3S_2/CC, 20% Pt/C/CC in 1.0 mol L^{-1} KOH electrolyte with (solid lines)/without (dot-dash lines) the addition of 1.0 mol L^{-1} methanol without *iR*-compensation.

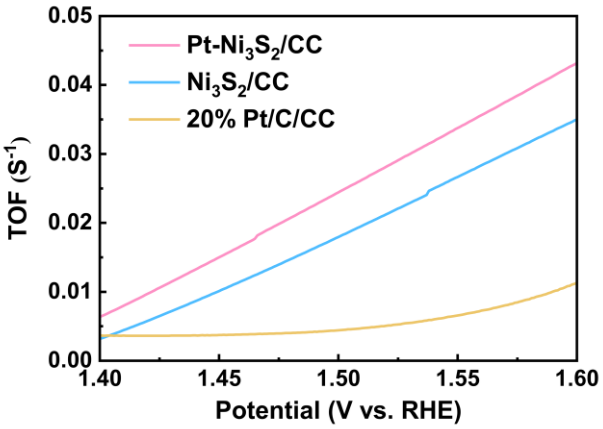

Fig. S8 The turnover frequency of the Pt-Ni_3S_2/CC, Ni_3S_2/CC, 20% Pt/C/CC catalysts calculated from MOR polarization curves in 1.0 mol L^{-1} KOH with 1.0 mol L^{-1} methanol.

The values of TOF were calculated by assuming that all metal atoms are involved in the catalytic processes, which all represent the lowest limits of the models:

$$TOF=j*S/zFn$$

where j (mA cm^{-2}) is the as-measured current density at various potentials, S (cm^{-2}) represents the surface area of the glassy carbon disk, the number z means a four-electron transfer during the formation of one mole of HCOOH for MOR (two-electron transfer during the formation of one mole of H_2 for HER), F is the Faraday's constant (96485.3 C mol^{-1}), and n is the moles of Ni atoms on the electrode which can be calculated by the loading weight and the molecular weight of the coated catalysts.

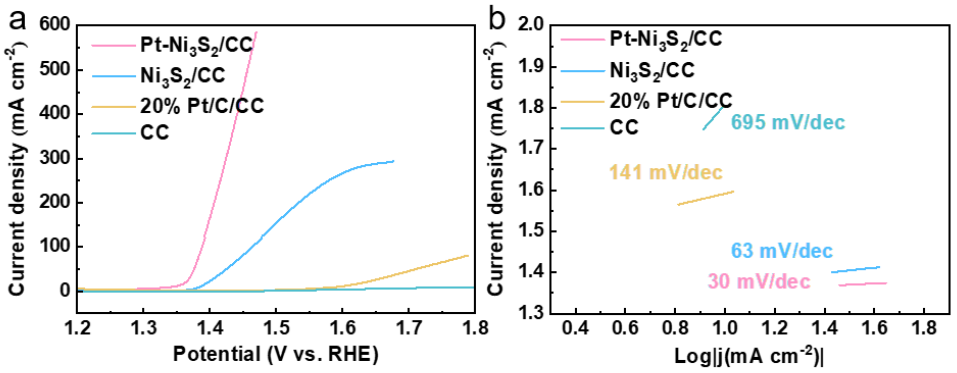

Fig. S9 (**a**) LSV curves and (**b**) Tafel slope of Pt-Ni_3S_2/CC, Ni_3S_2/CC, 20% Pt/C/CC and pristine carbon cloth (CC) in 1.0 mol L^{-1} KOH containing 1.0 mol L^{-1} methanol with *iR*-compensation.

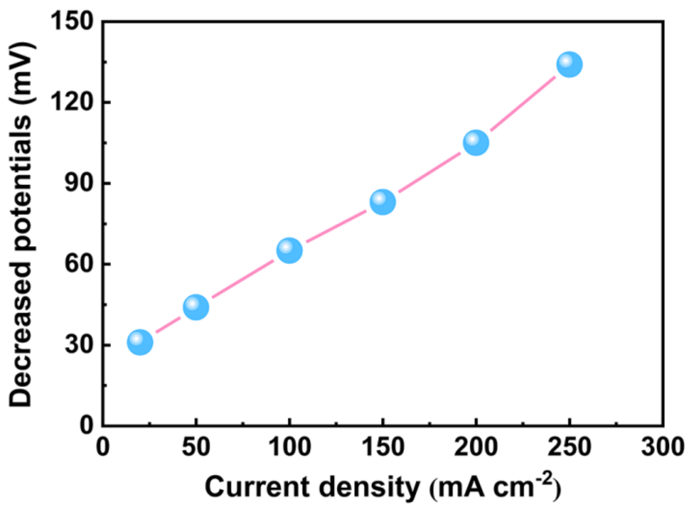

Fig. S10 The decreased oxidative potentials of methanol upgrading reaction using Pt-Ni_3S_2/CC electrode at certain current densities compared with Ni_3S_2/CC electrode

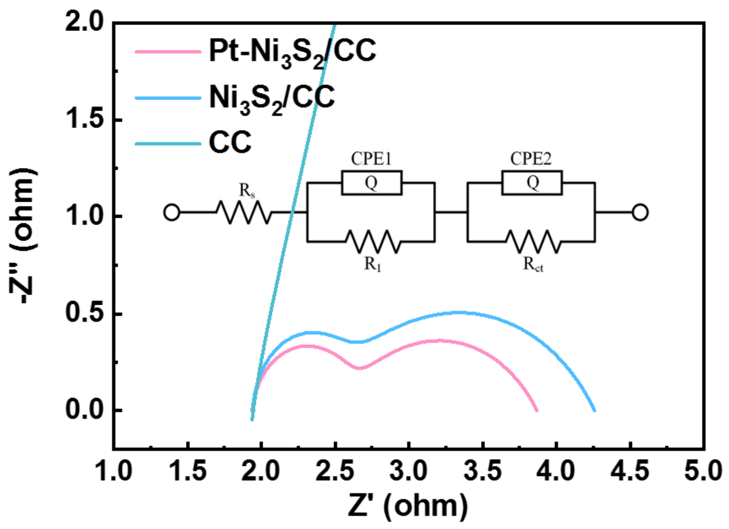

Fig. S11 EIS of Pt-Ni_3S_2/CC, Ni_3S_2/CC and pristine carbon cloth (CC) in 1.0 mol L^{-1} KOH containing 1.0 mol L^{-1} methanol

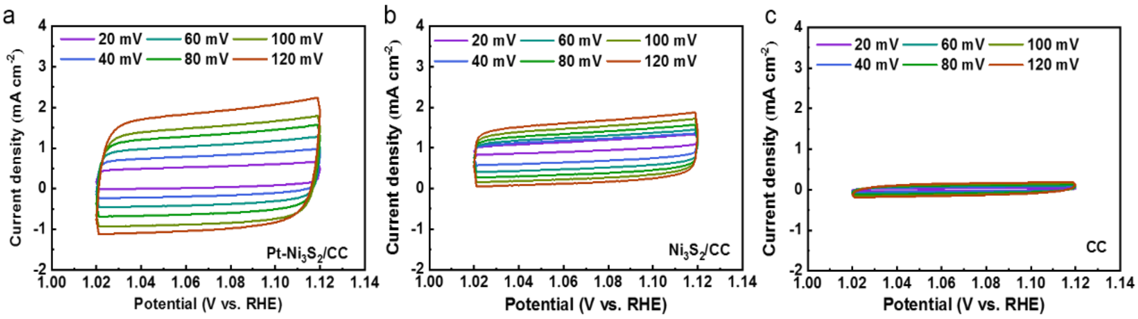

Fig. S12 Cyclic voltammetry curves of (**a**) Pt-Ni_3S_2/CC, (**b**) Ni_3S_2/CC, (**c**) pristine CC at different scan rates from 20 to 120 mV s^{-1} in 1.0 mol L^{-1} KOH containing 1.0 mol L^{-1} methanol

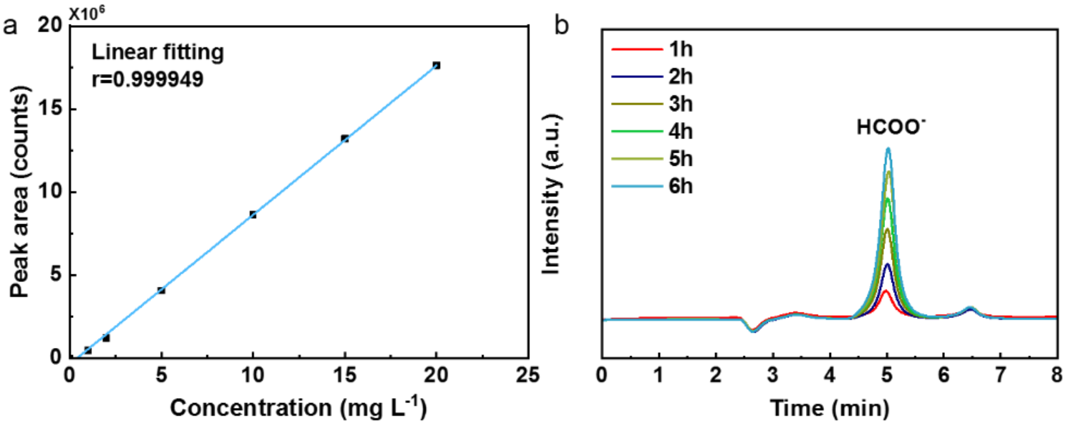

Fig. S13 (**a**) The establishment of formate calibration curve by linear fitting based on the IC chromatogram traces. (**b**) IC traces for detecting the formate concentration in the electrolyte (1.0 mol L^{-1} KOH + 1.0 mol L^{-1} formate) after the 6 hours' MOR with Pt-Ni_3S_2/CC anode

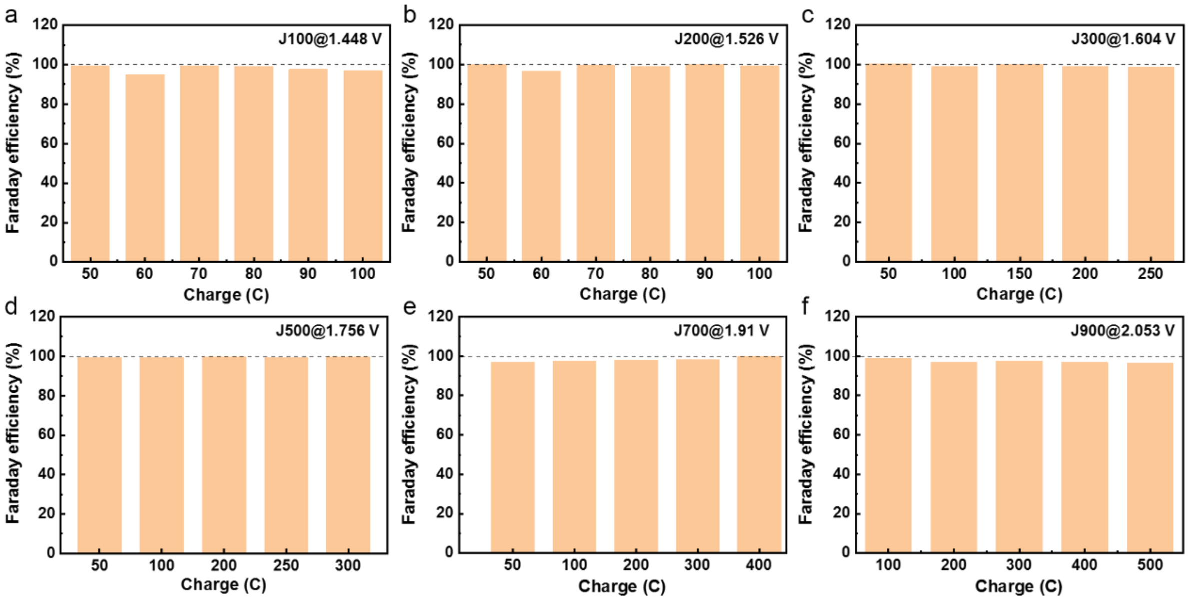

Fig. S14 Faraday efficiency at different voltages (**a**) 1.448 V at 100 mA cm^{-2}, (**b**) 1.526 V at 200 mA cm^{-2}, (**c**) 1.604 V at 300 mA cm^{-2}, (**d**) 1.756 V at 500 mA cm^{-2}, (**e**) 1.91 V at 700 mA cm^{-2}, (**f**) 2.053 V at 900 mA cm^{-2}

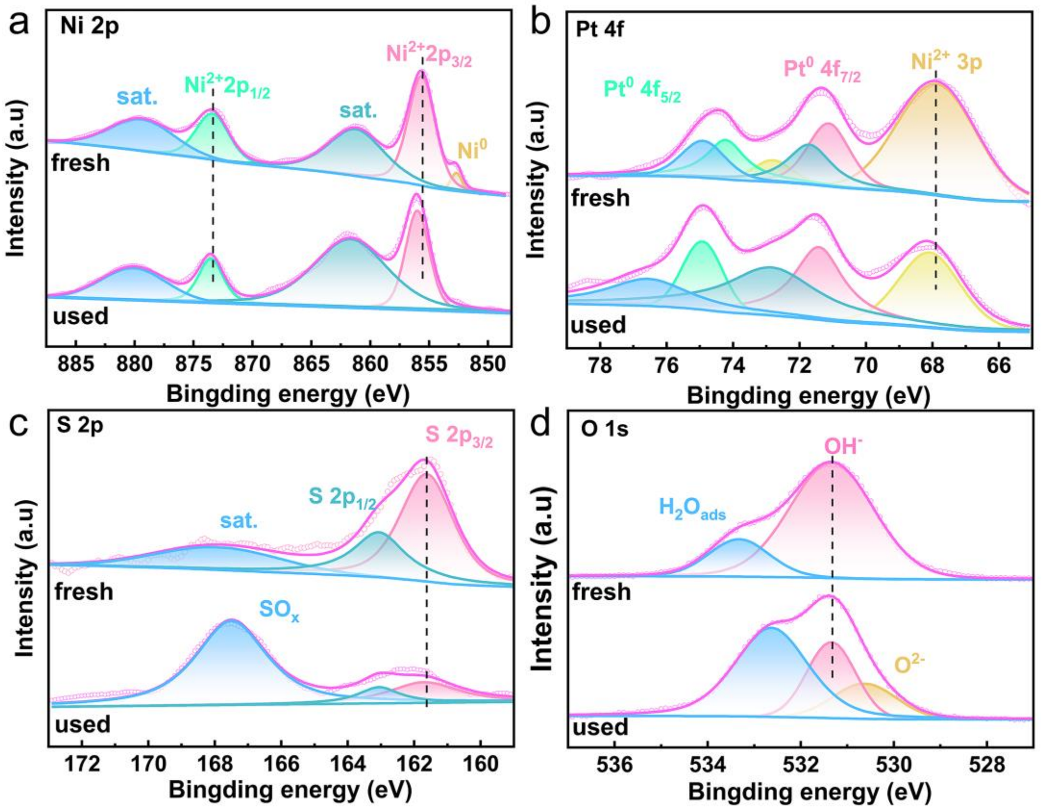

Fig. S15 High-resolution XPS spectra. The comparison of fresh and used Pt-Ni_3S_2 nano-heterostructures in (**a**) Ni 2p, (**b**) Pt 4f, (**c**) S 2p and (**d**) O 1s regions after MOR stability tests by chronoamperometry (*I*-*t*) at 2.12 V (vs. RHE) with an initial current density of ~1000 mA cm^{-2} for 72 h

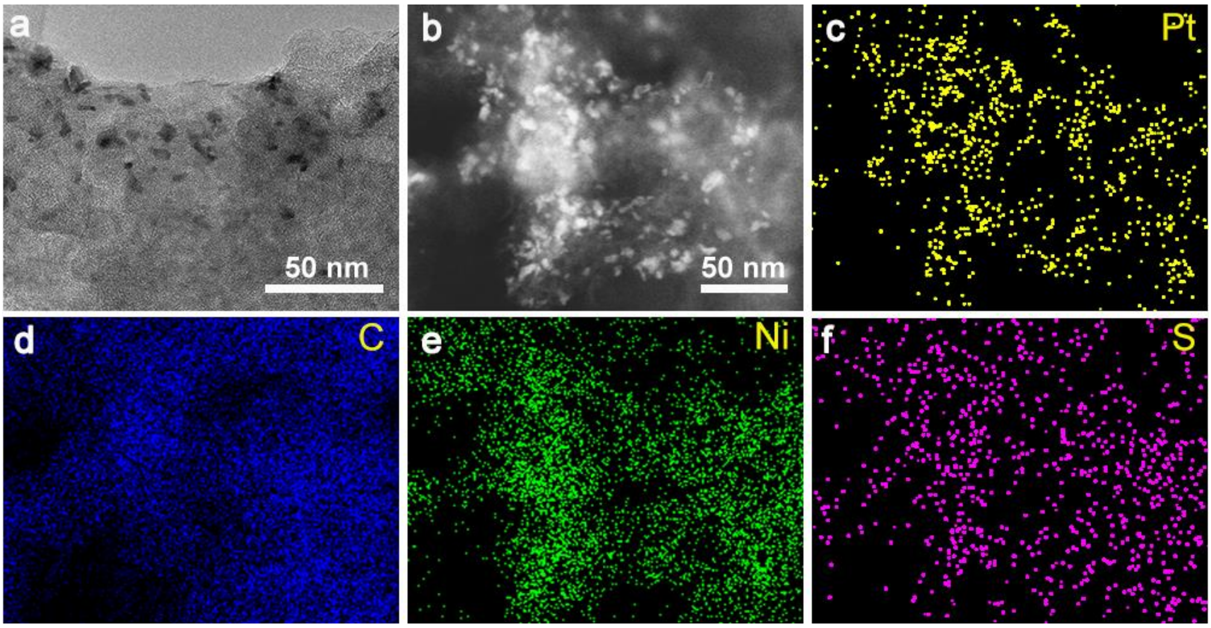

Fig. S16 (**a**) HRTEM images; (**b**) HAADF-STEM image and the corresponding EDS elemental (**c-f**) mapping images of Pt-Ni_3S_2 nano-heterostructures after MOR stability tests by chronoamperometry (*I*-*t*) at 2.12 V (vs. RHE) with an initial current density of ~1000 mA cm^{-2} for 72 h

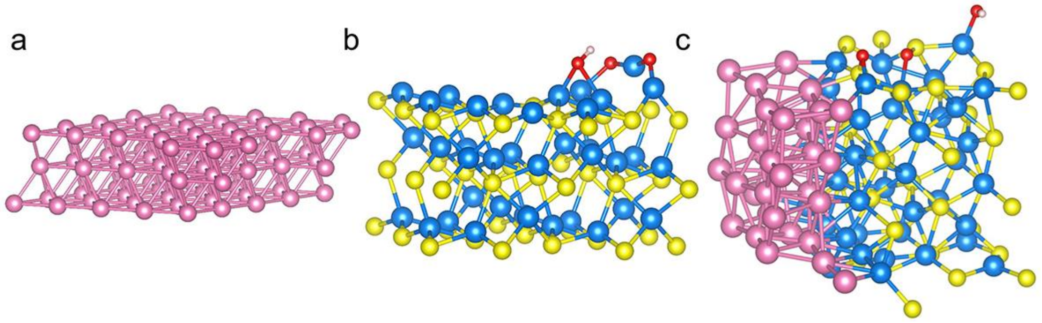

Fig. S17 The computational models of (**a**) Pt, (**b**) Ni_3S_2 and (**c**) Pt-Ni_3S_2 about MOR

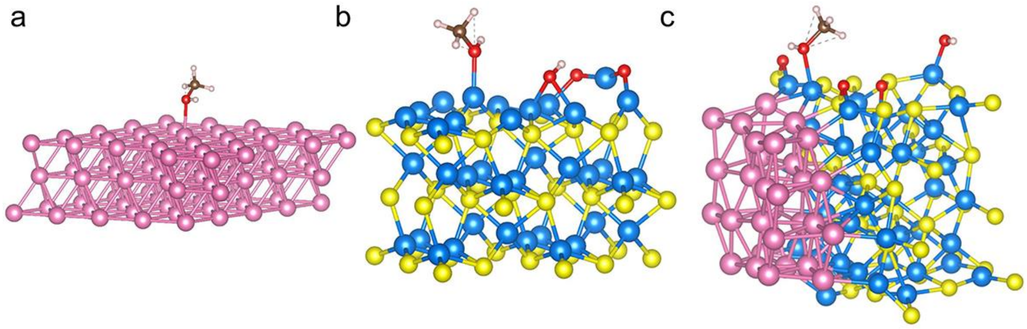

Fig. S18 CH_3OH binding models of (**a**) Pt, (**b**) Ni_3S_2 and (**c**) Pt-Ni_3S_2

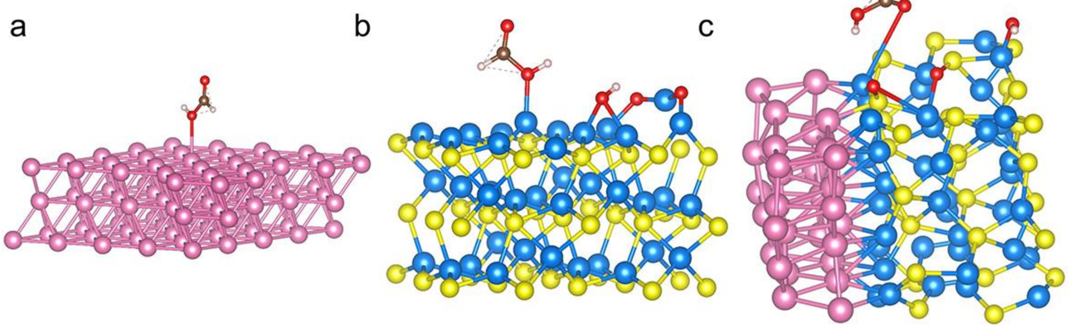

Fig. S19 HCOOH binding models of (**a**) Pt, (**b**) Ni_3S_2 and (**c**) Pt-Ni_3S_2

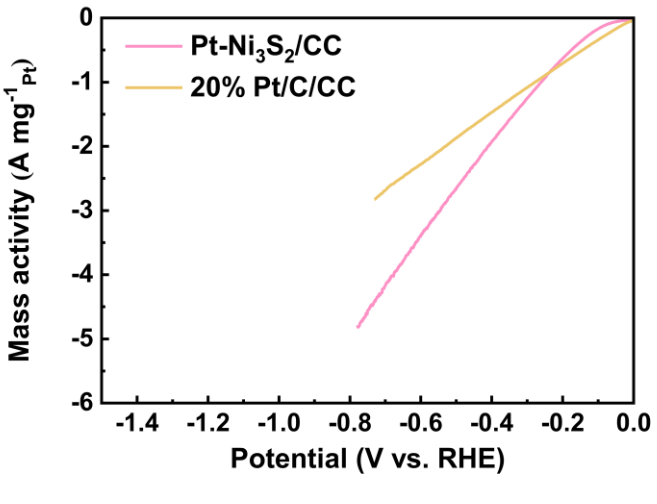

Fig. S20 The electrocatalytic performance about mass activity with Pt-Ni_3S_2/CC, and 20% Pt/C/CC

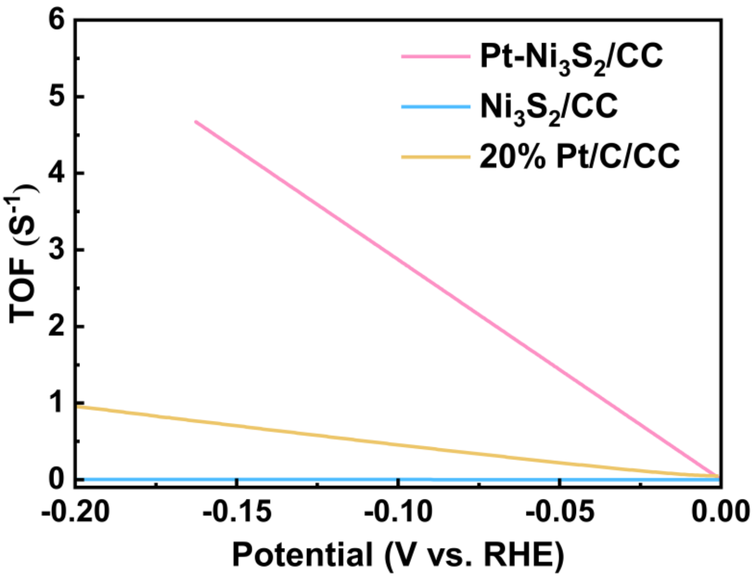

Fig. S21 The turnover frequency of the Pt-Ni_3S_2/CC, Ni_3S_2/CC, 20% Pt/C/CC catalysts calculated from HER polarization curves in 1.0 mol L^{-1} KOH

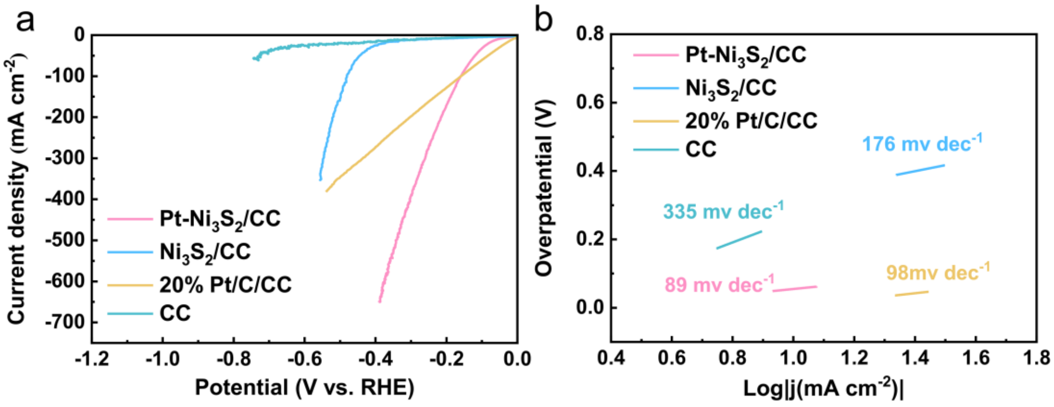

Fig. S22 (**a**) LSV curves and (**b**) Tafel slope plots of Pt-Ni_3S_2/CC, Ni_3S_2/CC, 20% Pt/C/CC and pristine carbon cloth (CC) in 1.0 mol L^{-1} KOH with *iR*-compensation

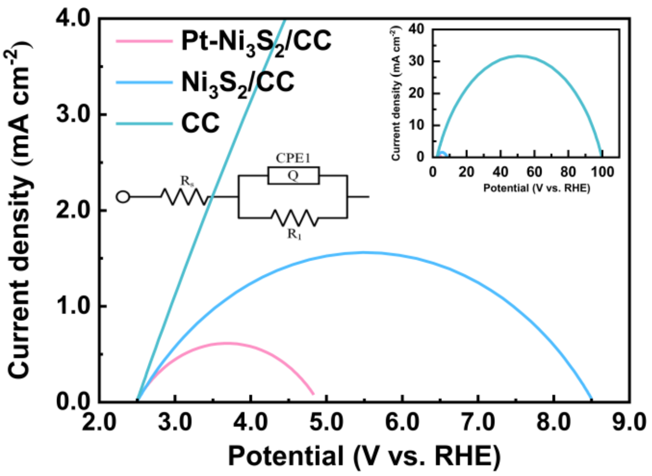

Fig. S23 EIS curves of Pt-Ni_3S_2/CC, Ni_3S_2/CC and pristine carbon cloth (CC) in 1.0 mol L^{-1} KOH

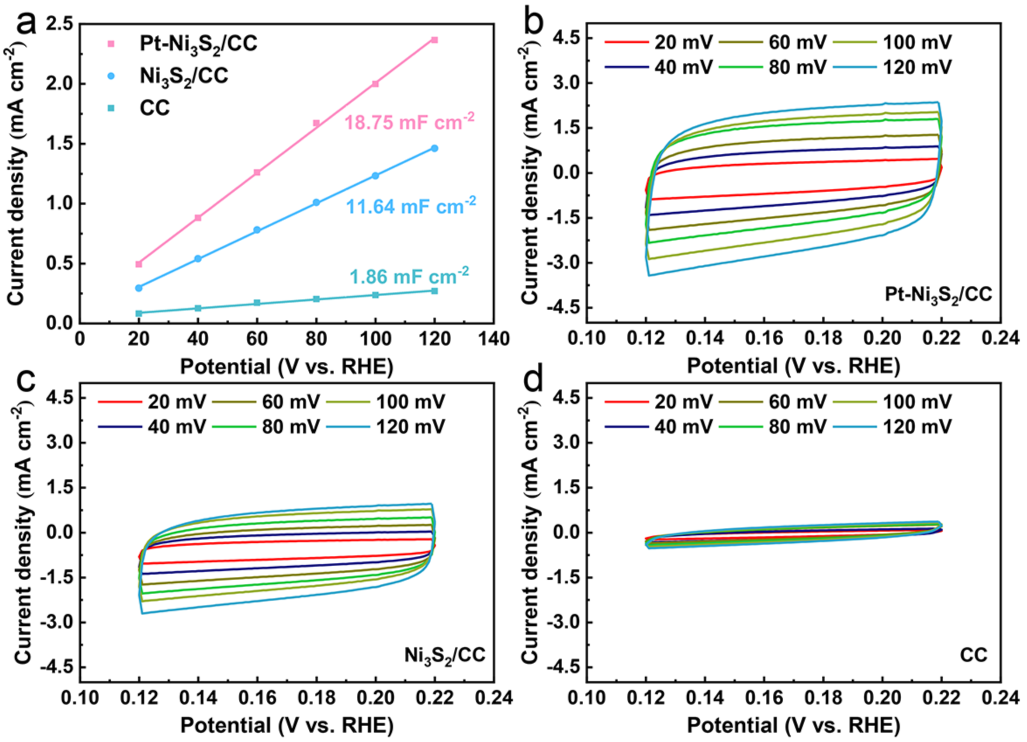

Fig. S24 (**a**) Calculated electrochemical double-layer capacitances (*Cdl*) of Pt-Ni_3S_2/CC, Ni_3S_2/CC, CC, Cyclic voltammetry curves of (**b**) Pt-Ni_3S_2/CC, (**c**) Ni_3S_2CC, (**d**) pristine CC at different scan rates from 20 to 120 mV s^{-1} in 1.0 mol L^{-1} KOH

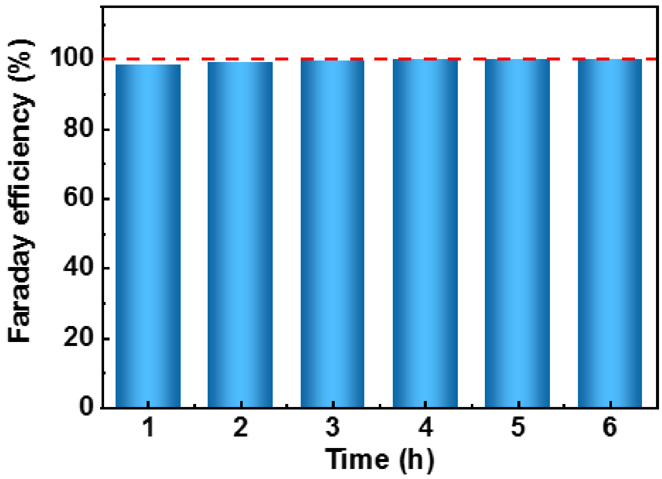

Fig. S25 The corresponding faradaic efficiencies for H_2 generation in 1.0 mol L^{-1} KOH

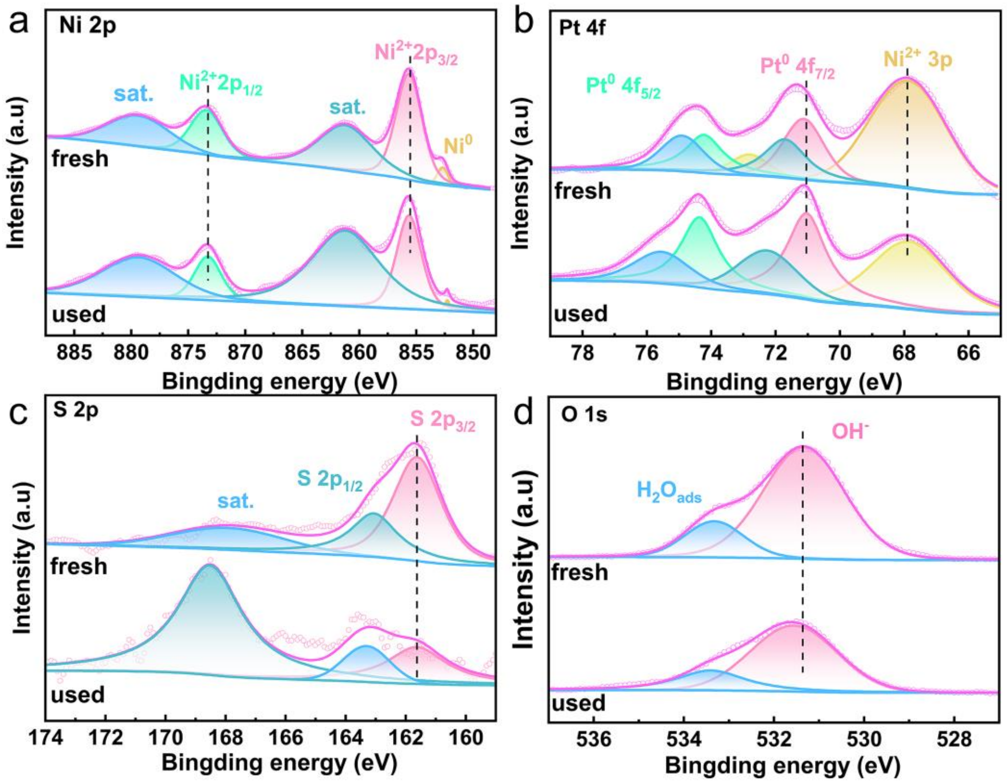

Fig. S26 High-resolution XPS spectra. The comparison of fresh and used Pt-Ni_3S_2 nano-heterostructures in (**a**) Ni 2p, (**b**) Pt 4f, (**c**) S 2p and (**d**) O 1s regions after HER stability tests by chronoamperometry (*I*-*t*) with current density of 100 mA cm^{-2} for 24 h

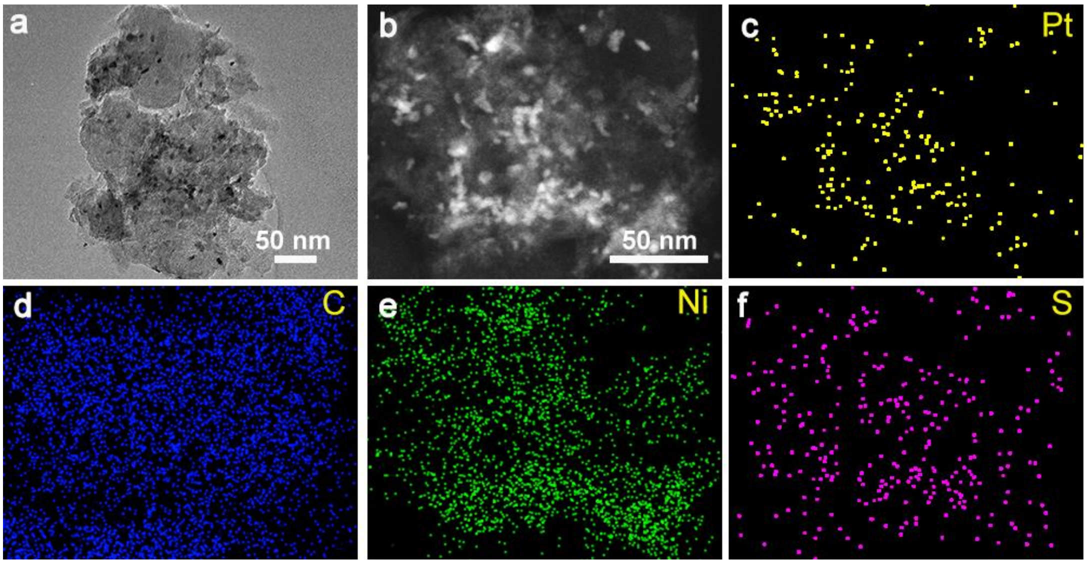

Fig. S27 (**a**) HRTEM images; (**b**) HAADF-STEM image and the corresponding EDS elemental (**c-f**) mapping images of Pt-Ni_3S_2 nano-heterostructures after HER stability tests by chronoamperometry (*I*-*t*) with current density of 100 mA cm^{-2} for 24 h

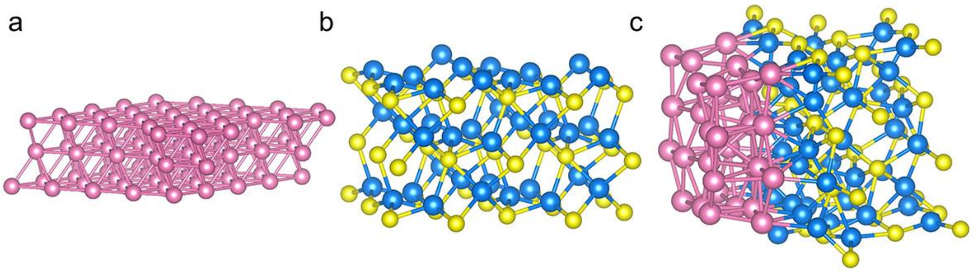

Fig. S28 The computational models of (**a**) Pt, (**b**) Ni_3S_2 and (**c**) Pt-Ni_3S_2 about HER

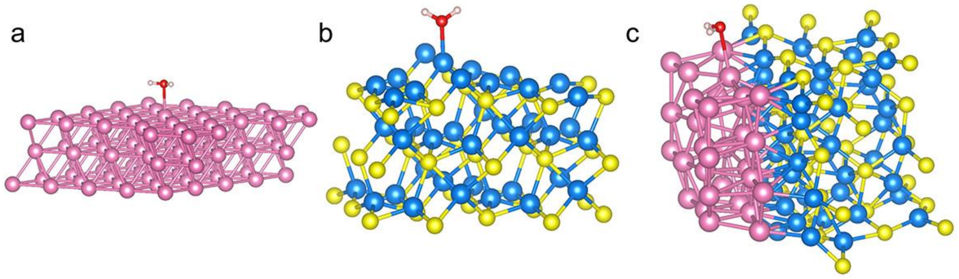

Fig. S29 H_2O binding models of (**a**) Pt, (**b**) Ni_3S_2 and (**c**) Pt-Ni_3S_2

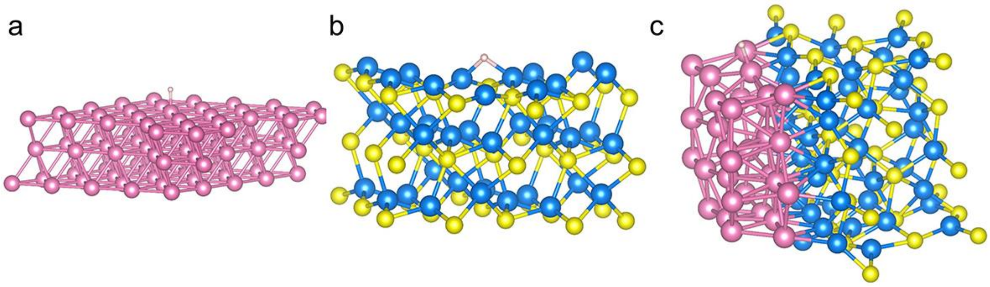

Fig. S30 H binding models of (**a**) Pt, (**b**) Ni_3S_2 and (**c**) Pt-Ni_3S_2

3 Supplementary Tables

Table S1 Element content analysis of Pt-Ni_3S_2 via ICP-MS.

**Element**	Mass Fraction (%)	Atom ratio
Ni	69.67	1.18
Pt	9.38	0.05
S	20.96	0.66

Table S2 Fitting impedance parameters of various catalysts for MOR

**Catalysts**	R_s (Ω)	R_{ct1} (Ω)	R_{ct2} (Ω)
Pt-Ni_3S_2/CC	2.391	0.634	1.297
Ni_3S_2/CC	1.936	0.529	1.795

Table S3 Fitting impedance parameters of various catalysts for HER

**Catalysts**	R_s (Ω)	R_{ct} (Ω)
Pt-Ni_3S_2/CC	2.392	2.408
Ni_3S_2/CC	2.494	6.031

Table S4 MOR activities of Pt-Ni_3S_2 compared with those of Pt-based, Ni-based and S-based materials reported previously.

	**Electrocatalyst**	**Electrolyte**	**Potential@10 mA cm^{-2} (V vs. RHE)**	**Potential@100 mA cm^{-2} (V vs. RHE)**	**Peak current density, mass activity, or stability test**	**Refs.**
Ni-based	**Pt-Ni_3S_2**	**1.0 M KOH + 1.0 M MeOH**	**1.35**	**1.448**	**> 700 mA cm^{-2} (equivalent to > 5.18 A mg^{-1}) after 72 hours' reaction by *I-t***	**This work**
	Ni_3C	1.0 M KOH + 1.0 M MeOH	~1.39	~1.6		[S3]
	NiP_x-R	1.0 M KOH + 0.5 M MeOH	~1.36	1.49		[S4]
	NiS_x-R	1.0 M KOH + 0.5 M MeOH	~1.362	~1.51		[S4]
	$NiSe_x$-R	1.0 M KOH + 0.5 M MeOH	~1.38	~1.53		[S4]
	$Ni_{0.75}Fe_{0.25}Se_2$	1.0 M KOH + 0.5 M MeOH	1.40	~1.58		[S5]
	NiB-400	1.0 M KOH + 1.0 M MeOH	~1.38	~1.51		[S6]
	CuONS/CF	1.0 M KOH + 1.0 M MeOH	~1.37	1.50		[S7]
	Ni_3B/Ni	1.0 M KOH + 1.0 M MeOH	~1.38	1.526		[S8]
	Ni polyhedral NCs	1.0 M KOH + 1.0 M MeOH	~1.5	~1.6		[S9]
Pt-based	Pt_1/RuO_2	0.1 M KOH + 1.0 M MeOH			6.766 A mg^{-1} from CV/LSV at 50 mV s^{-1}, absence of *I-t* or CP tests	[S10]
	Pt/Ni$(OH)_2$/rGO	1.0 M KOH + 1.0 M MeOH			< 0.2 A mg^{-1} after 55.56 hours' reaction by *I-t*	[S11]
	NiPtSAA/GDY	1.0 M KOH + 1.0 M MeOH			< 25 mA cm^{-2} after 20 hours' reaction by *I-t*	[12]
	Pt_3Bi_3Zn	0.1 M $HClO_4$ + 0.5 M MeOH			3.29 A mg^{-1} from CV/LSV at 20 mV s^{-1}, absence of *I-t* or CP tests	[S13]
	Pt NPs traped by CNTs	0.5 M H_2SO_4 + 1.0 M MeOH			0.149 A mg^{-1} from CV/LSV at 50 mV s^{-1}, < 0.002 mA	[S14]

					cm^{-2} after 0.5 hour's reaction by *I-t*	
	Pt/bTNT5	0.1 M $HClO_4$ + 0.5 M MeOH			< 70 mA cm^{-2} after 1 hour's reaction by *I-t*	[S15]
S-based	$Ni(OH)_2Ni_3S_4$	1.0 M KOH + 0.5 M MeOH		1.61		[S16]
	NiS_x-R	1.0 M KOH + 0.5 M MeOH	~1.362	~1.51		[S4]

Table S5 HER activities of Pt-Ni_3S_2 should be compared with those of Pt-based, Ni-based and S-based materials reported previously

	**Electrocatalyst**	**Electrocatalyst**	**Potential@10 mA cm-2 (V vs. RHE)**	**Refs.**
Ni-based	**Pt-Ni_3S_2**	**1.0 M KOH**	**60**	**This work**
	Au/Ni_3S_2	1.0 M KOH	97	[S17]
	Ni_3S_2/MnS-O	1.0 M KOH	116	[S18]
	$CoNi_2S_4$/Ni_3S_2	1.0 M KOH	171	[S19]
	δ-FeOOH/Ni_3S_2	1.0 M KOH	106	[S20]
	NC@NiNPs	1.0 M KOH	74	[S21]
Pt-based	Pt_4/s-MoO_3	1.0 M KOH	123	[S22]
	$Pt_{85}Mo_{15}$-Se NWs	0.1 M NaOH	>> 100 mV	[S23]
S-based	$NiWO_4$-Ni_3S_2	1.0 M KOH	136	[S24]
	Fe-Mo-S/Ni_3S_2	1.0 M KOH	141	[S25]

S4 Equations

In 1.0 mol L^{-1} KOH with 1.0 mol L^{-1} methanol, the equations describe the anode, cathode and overall electrochemical reactions are proposed as follows, respectively.

$$\text{Anode:} \quad CH_3OH + 5OH^- \rightarrow HCOO^- + 4H_2O + 4e^- \qquad \text{(Eq. S1)}$$

$$\text{Cathode:} \quad 4H_2O + 4e^- \rightarrow 2H_2\uparrow + 4OH^- \qquad \text{(Eq. S2)}$$

$$\text{The overall:} \quad CH_3OH + OH^- \rightarrow HCOO^- + 2H_2\uparrow \qquad \text{(Eq. S3)}$$

In the alkaline electrolyte, the formation of one mole formate will consume five-fold amount of hydroxyl ions in the anode compartment (Equation S1), which is a four-electron transfer process. Accordingly, four moles of electrons will react with 4 moles of water molecules at the cathode to form 2 moles of H_2 gas and 4 moles of hydroxyl ions (Equation S2).

Since the generation of formate in alkaline environment will consume the hydroxyl ions, the pH of the electrolyte will be decreased along with the production of formate. The results also indicate that the high alkaline concentration is beneficial for the high reactivity of methanol upgrading reaction.

Supplementary References

[S1] S. Niu, S. Li, Y. Du, X. Han, P. Xu. How to reliably report the overpotential of an electrocatalyst. ACS Energy Lett. **5**, 1083-1087 (2020). https://doi.org/10.1021/acsenergylett.0c00321

[S2] L. H. Sun, Q. Y. Li, S. N. Zhang, D. Xu, Z. H. Xue et al. Heterojunction-based electron donators to stabilize and activate ultrafine Pt nanoparticles for efficient hydrogen atom dissociation and gas evolution. Angew. Chem. Int. Ed. **60**, 25766-25770 (2021). https://doi.org/10.1002/anie.202111920

[S3] J. S. Li, R. L. Wei, X. Wang, Y. Zuo, X. Han et al. Selective methanol-to-formate electrocatalytic conversion on branched nickel carbide. Angew. Chem. Int. Ed. **59**, 20826-20830 (2020). https://doi.org/10.1002/anie.202004301

[S4] S. L. Li, R. G. Ma, J. C. Hu, Z. C. Li, L. J. Liu et al. Coordination environment tuning of nickel sites by oxyanions to optimize methanol electro-oxidation activity. Nat. Commun. **13**, 2916 (2022). https://doi.org/10.1038/s41467-022-30670-4

[S5] J. Li, C. Xing, Y. Zhang, T. Zhang, M. C. Spadaro et al. Nickel iron diselenide for highly efficient and selective electrocatalytic conversion of methanol to formate. Small **17**, e2006623 (2021). https://doi.org/10.1002/smll.202006623

[S6] Y. B. Qi, Y. Zhang, L. Yang, Y. H. Zhao, Y. H. Zhu et al. Insights into the activity of nickel boride/nickel heterostructures for efficient methanol electrooxidation. Nat. Commun. **13**, 4602 (2022). https://doi.org/10.1038/s41467-022-32443-5

[S7] X. F. Wei, Y. Li, L. S. Chen, J. L. Shi. Formic acid electro-synthesis by concurrent cathodic CO_2 reduction and anodic ch_3oh oxidation. Angew. Chem. Int. Ed. **60**, 3148-3155 (2021). https://doi.org/10.1002/anie.202012066

[S8] Z. Liu, P. Chang, M. Xi, J. Ding, X. Wang et al. Synthesis of Ni(3) B/Ni via vacuum-induced for ultrahigh stable and efficient methanol oxidation. Small 2303855 (2023). https://doi.org/10.1002/smll.202303855

[S9] J. Li, Y. Zuo, J. Liu, X. Wang, X. Yu et al. Superior methanol electrooxidation performance of (110)-faceted nickel polyhedral nanocrystals. J. Mater. Chem. A **7**,

22036-22043 (2019). https://doi.org/10.1039/c9ta07066d

[S10] Z. Q. Zhang, J. P. Liu, J. Wang, Q. Wang, Y. H. Wang et al. Single-atom catalyst for high-performance methanol oxidation. Nat. Commun. **12**, 5235 (2021). https://doi.org/10.1038/s41467-021-25562-y

[S11] W. Huang, H. Wang, J. Zhou, J. Wang, P. N. Duchesne et al. Highly active and durable methanol oxidation electrocatalyst based on the synergy of platinum-nickel hydroxide-graphene. Nat. Commun. **6**, 10035 (2015). https://doi.org/10.1038/ncomms10035

[S12] L. Hui, Y. R. Xue, C. Y. Xing, Y. X. Liu, Y. C. Du et al. Atomic alloys of nickel-platinum on carbon network for methanol oxidation. Nano Energy **95**, 106984 (2022). https://doi.org/10.1016/j.nanoen.2022.106984

[S13] H. Tian, D. Wu, J. Li, J. Luo, C. Jia et al. Rational design ternary platinum based electrocatalysts for effective methanol oxidation reaction. J. Energy Chem. **70**, 230-235 (2022). https://doi.org/10.1016/j.jechem.2022.02.021

[S14] P. R. M, S. Berchmans. Electrochemically activated platinum nanoparticles trapped by carbon nanotubes for methanol oxidation reaction. J. Electroanal. Chem. **940**, 117492 (2023). https://doi.org/10.1016/j.jelechem.2023.117492

[S15] A. Touni, X. Liu, X. Kang, C. Papoulia, E. Pavlidou et al. Methanol oxidation at platinum coated black titania nanotubes and titanium felt electrodes. Molecules. **27**, 6382 (2022). https://doi.org/10.3390/molecules27196382

[S16] C. Hou, W. Yang, X. Yang, B. Li, H. Gao et al. In situ sulfidation for controllable hetero-interface engineering of α-$Ni(OH)_2$-Ni_3S_4 hybrid structures realizing robust electrocatalytic methanol oxidation. Chem. Commun. **56**, 5283-5286 (2020). https://doi.org/10.1039/d0cc01298j

[S17] H. Liu, J. N. Cheng, W. J. He, Y. Li, J. Mao et al. Interfacial electronic modulation of Ni_3S_2 nanosheet arrays decorated with au nanoparticles boosts overall water splitting. Appl. Catal. B Environ. **304**, 120935 (2022). https://doi.org/10.1016/j.apcatb.2021.120935

[S18] Y. Zhang, J. Fu, H. Zhao, R. Jiang, F. Tian et al. Tremella-like Ni_3S_2/mns with ultrathin nanosheets and abundant oxygen vacancies directly used for high speed overall water splitting. Appl. Catal. B Environ. **257**, 117899 (2019). https://doi.org/10.1016/j.apcatb.2019.117899

[S19] W. Dai, K. Ren, Y.-a. Zhu, Y. Pan, J. Yu et al. Flower-like $CoNi_2S_4$/Ni_3S_2 nanosheet clusters on nickel foam as bifunctional electrocatalyst for overall water splitting. J. Alloys Compd. **844**, 156252 (2020). https://doi.org/10.1016/j.jallcom.2020.156252

[S20] X. Ji, C. Cheng, Z. Zang, L. Li, X. Li et al. Ultrathin and porous δ-FeOOH modified Ni_3S_2 3D heterostructure nanosheets with excellent alkaline overall water splitting performance. J. Mater. Chem. A **8**, 21199-21207 (2020). https://doi.org/10.1039/d0ta07676g

[S21] T. Yang, L. Fan, L. Wenqian, M. Shaoli, L. Xiaohong et al. Multifunctional carbon-

armored ni electrocatalyst for hydrogen evolution under high current density in alkaline electrolyte solution. Appl. Catal. B Environ. **321**, 122081 (2022). https://doi.org/10.1016/j.apcatb.2022.122081

[S22] T. Peng, H. Po-Yuan, E. N. S. Jack, W. Chenbo, G. Diego et al. Structure–property relationship of defect-trapped Pt single-site electrocatalysts for the hydrogen evolution reaction. ACS Catal. **13**, 9558-9566 (2023). https://doi.org/10.1021/acscatal.3c01513

[S23] L. Yu, T. Zhou, S. Cao, X. Tai, L. Liu et al. Suppressing the surface passivation of Pt-Mo nanowires via constructing Mo-Se coordination for boosting her performance. Nano Res. **1-7**, (2020). https://doi.org/10.1007/s12274-020-3269-8

[S24] S. Huang, Y. Meng, Y. Cao, F. Yao, Z. He et al. Amorphous $NiWO_4$ nanoparticles boosting the alkaline hydrogen evolution performance of Ni_3S_2 electrocatalysts. Appl. Catal. B Environ. **274**, 119120 (2020). https://doi.org/10.1016/j.apcatb.2020.119120

[S25] Y. Zhang, H. Guo, X. Li, J. Du, W. Ren et al. A 3D multi-interface structure of coral-like Fe-Mo-S/Ni_3S_2@NF using for high-efficiency and stable overall water splitting. Chem. Eng. J. **404**, 126483 (2020). https://doi.org/10.1016/j.cej.2020.126483
